# C-5’-Triazolyl-2’-oxa-3’-aza-4’a-carbanucleosides: Synthesis and biological evaluation

**DOI:** 10.3762/bjoc.11.38

**Published:** 2015-03-09

**Authors:** Roberto Romeo, Caterina Carnovale, Salvatore V Giofrè, Maria A Chiacchio, Adriana Garozzo, Emanuele Amata, Giovanni Romeo, Ugo Chiacchio

**Affiliations:** 1 Dipartimento Scienze del Farmaco e dei Prodotti per la Salute, University of Messina, Via S.S. Annunziata, 98168 Messina, Italy; 2 Dipartimento di Scienze del Farmaco, University of Catania, Via A. Doria 6, 95125-Catania, Italy; 3 Dipartimento di Scienze Bio-Mediche, University of Catania,Via Androne 81, 95124 Catania, Italy

**Keywords:** antitumor activity, click chemistry, 1,3-dipolar cycloaddition, nucleic acids, 2’-oxa-3’-aza-4’a-carbanucleoside analogs

## Abstract

A novel series of 2’-oxa-3’-aza-4’a-carbanucleosides, featured with a triazole linker at the 5’-position, has been developed by exploiting a click chemistry reaction of 5’-azido-2’-oxa-3’-aza-4’a-carbanucleosides with substituted alkynes. Biological tests indicate an antitumor activity for the synthesized compounds: most of them inhibit cell proliferation of Vero, BS-C-1, HEp-2, MDCK, and HFF cells with a CC_50_ in the range of 5.0–40 μM. The synthesized compounds do not show any antiviral activity.

## Introduction

Synthetic modified nucleosides are of great interest as potential new lead structures in particular as antiviral or anticancer agents [1–8]. As analogues these compounds can interfere in nucleic acid synthesis or block nucleosides- and/or nucleotide-dependent biological processes by mimicking natural nucleosides and serving as inhibitors or building units [9–12]. Many structural variations of the natural nucleosides have been exploited. In general, the performed modifications included the replacement of the furanose moiety by other carbon or heterocyclic systems [13–14] or even acyclic fragments [15–16], the substitution of pyrimidine or purine natural nucleobases with unnaturally-substituted heteroaromatics or homoaromatic systems, or the modification of the phosphate P(O)–O–C bond with the non–hydrolyzable phosphonate P(O)–C linkage [17–18].

**Figure 1:**
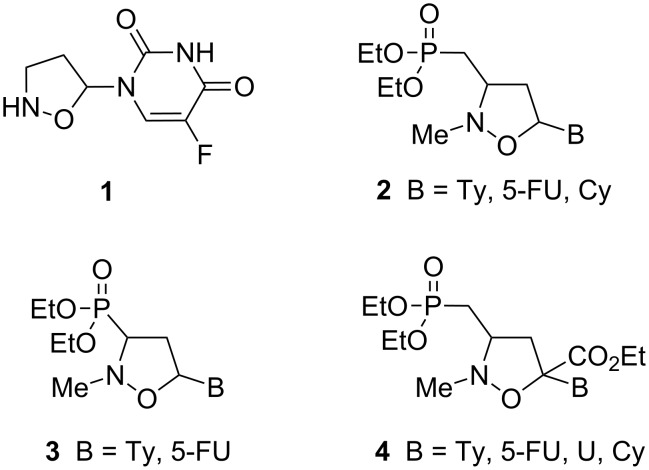
2’-Oxa-3’-aza-modified nucleosides and 2’-oxa-3’-aza-modified nucleotides.

In this context, nucleoside analogues, where different carbon or heterocyclic systems replace the furanose ring, have been reported as anticancer or antiviral agents [19–20]. In particular, 2’-oxa-3’-aza-4’a-carbanucleosides **1**–**4**, characterized by the presence of an isoxazolidine ring, represent a scaffold of modified dideoxynucleosides endowed with interesting physiological features (Figure 1) [21–27].

2’-Oxa-3’-aza-4’a-carbanucleosides **1–4** can be considered as mimics of natural nucleosides and act as terminators of the viral DNA chain. Their antiviral activity is linked to the competitive reversible inhibition of the reverse transcriptase. Furthermore, as antimetabolites, they can interact with intracellular targets to induce cytotoxicity [28–32].

Several functionalities have been inserted as linkers on the 2’-oxa-3’-aza-4’a-carbanucleoside skeleton in order to confer novel mechanisms of action for nucleoside mimics: in this context, the 1,2,3-triazole unit assumes particular interest according to its easily access and the well-known biological activity of many derivatives. In these last years, in fact, triazoles have gained considerable attention in medicinal chemistry, bioconjugation, drug-delivery, and materials science [33–38]. Moreover, the 1,2,3-triazole motif is exceedingly stable to basic or acidic hydrolysis and interacts strongly with biological targets through hydrogen bonding to nitrogen atoms as well as through dipole–dipole and π-stacking interactions [39].

**Figure 2:**
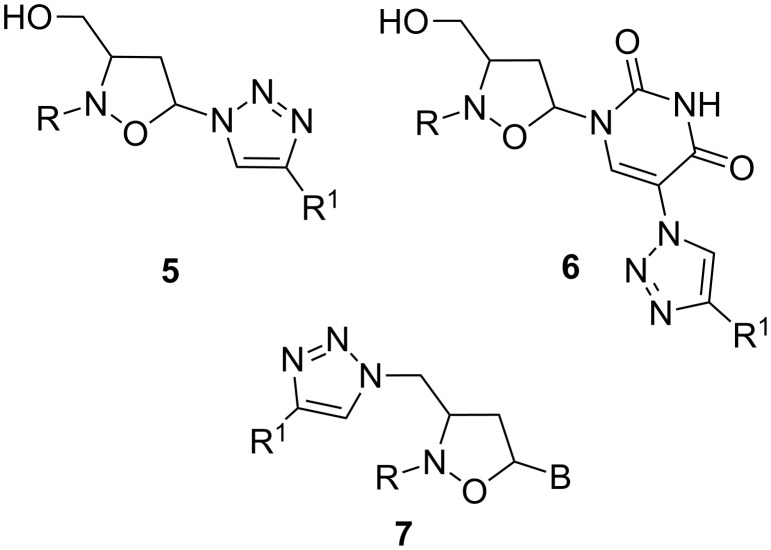
Triazolyl-2’-oxa-3’-aza-4’a-carbanucleosides.

Recently, a synthetic approach towards 3-hydroxymethyl-5-(1*H*-1,2,3-triazol)-isoxazolidines **5** has been described [40]: the obtained compounds inhibit the growth of anaplastic and follicular human thyroid cancer cell lines, with IC_50_ values in the range of 3.87–8.76 μM. In the same context, novel 1,2,3-triazole-appended 2’-oxa-3’-azanucleoside analogs **6** were developed [41]: Some of these compounds show a good anticancer activity against the anaplastic (8305C) and the follicular (FTC-133) human thyroid cancer cell lines, and especially on the U87MG human primary glioblastoma cell line (Figure 2).

Accordingly, considering that the incorporation of the triazole moiety can lead to interesting biological properties, we report in this paper the preparation of a small library of nucleoside analogues **7** (Figure 2), where the furanose ring is substituted by an isoxazolidine system and a triazole unit replaces the phosphodiester linker at 5’ position of the 2’-oxa-3’-aza-4’a-carbanucleoside. However, in order to maintain the six-bond periodicity of the oligonucleotides and thus the flexibility of the oligonucleotide chain the methylene bridge at the pseudo-5’-position was retained. The obtained compounds have shown to be endowed with an interesting antitumor activity: most of them inhibit cell proliferation of Vero, BS-C-1**,** HEp-2, MDCK, and HFF cells by 50% (CC_50_) at concentrations in the range of 5.0–40.0 μM. No antiviral activity against both RNA and DNA viruses was observed.

## Results and Discussion

### Chemistry

**Scheme 1:**
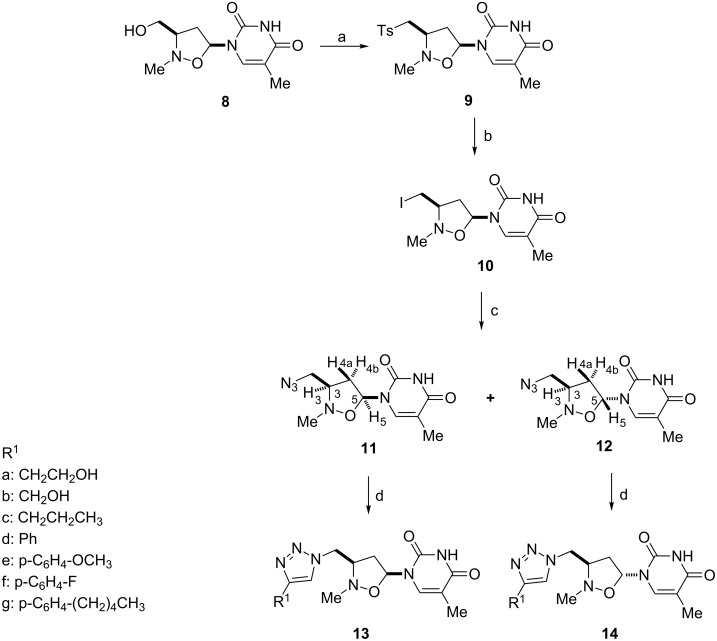
Synthesis of triazolyl isoxazolidinyl-nucleosides **13** and **14**. Reagents and conditions: a) Tosyl chloride, TEA, CH_2_Cl_2_, rt, 24 h; b) NaI, acetone, reflux, 72 h; c) NaN_3_, CH_3_CN/H_2_O (1:10) in the presence of NH_4_Cl, 50 °C for 48 h; d) substituted alkynes, **17a**–**g**, CuSO_4_·5H_2_O, sodium ascorbate, TEA, rt, 5 h.

The synthetic route to 5’-triazolyl-2’-oxa-3’-aza-4’a-carbanucleosides **13** and **14** is described in Scheme 1 (and Table 1). (3′*RS,*5′*SR*)-2′-*N*-methyl-3′-hydroxymethyl-1′,2′-isoxazolidin-5′-ylthymine **8**, obtained as the main compound, in a two-step process, by 1,3-dipolar cycloaddition of vinyl acetate to *C*-[(*tert*-butyldiphenylsilyl)oxy]-*N*-methylnitrone, followed by Hilbert–Jones nucleosidation using silylated thymine and TBAF [42–44], was converted into the corresponding iodo-derivative **10** by sequential tosylation and iodination.

**Table 1:** C-5’-Triazolyl-2’-oxo-3’-aza-4’a-carbanucleosides **13a–g** and **14a–g** produced via click chemistry.

Alkyne	R^1^	Product	Yield^a^	Product	Yield^a^

**17a**	–CH_2_CH_2_OH	**13a**	88	**14a**	79
**17b**	–CH_2_OH	**13b**	84	**14b**	81
**17c**	–CH_2_CH_2_CH_3_	**13c**	80	**14c**	83
**17d**	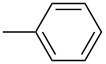	**13d**	78	**14d**	82
**17e**	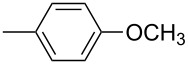	**13e**	78	**14e**	82
**17f**	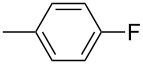	**13f**	85	**14f**	84
**17g**	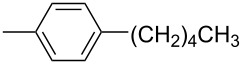	**13g**	89	**14g**	85

^a^Isolated yield by flash chromatography.

**Figure 3:**
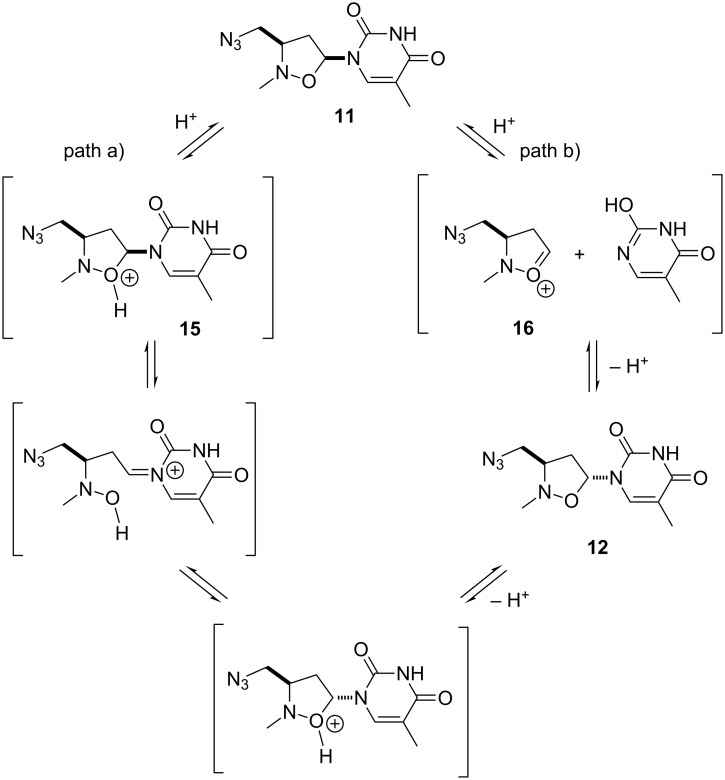
α–β Epimerization.

The subsequent reaction of **10** with sodium azide, performed at 50 °C in CH_3_CN/H_2_O (1:10) in the presence of NH_4_Cl for 48 h afforded two azides, **11** and **12**, epimeric at C-5, in a relative ratio 2:1 with a global yield of 85%. Two azides were separated by flash chromatography (CH_2_Cl_2_/MeOH 98:2 as eluent). Compound **12** originates from **11**: its formation can be rationalized by considering that the acidic medium of the reaction, linked to the presence of NH_4_Cl, promotes an equilibrium process which starts from **11** and leads to a mixture of α- and β-anomers, via the intermediate oxonium ion **15** (path a) or **16** (path b) (Figure 3). As reported in similar systems [45], in the equilibrium mixture the β-anomer **11**, thermodynamically more stable, predominates.

The structure of the obtained compounds was determined by spectroscopic data and MS analysis: the main product of the reaction was the *cis* derivative. NOE measurements confirm the assigned stereochemistry. For compound **11**, the *cis* isomer, irradiation of the H-5 resonance at 5.99 ppm (as doublet of doublets) induced a positive NOE effect on H-3 resonance at 3.85–4.00 ppm (as a multiplet) and on H-4b proton (2.34–2.42 ppm, multiplet) (Scheme 1). Accordingly, in the *trans* derivative **12**, on irradiating H-5 resonance (6.14 ppm; doublet of doublets), a positive NOE effect was detected only for the H-4a proton that resonates at 2.18 ppm as a doublet of doublet of doublets.

5’-Azido-2’-oxa-3’-aza-4’a-carbanucleosides **11** and **12** were independently engaged in a CuI-catalyzed Huisgen [3 + 2] cycloaddition reaction with a series of substituted alkynes **17**, according to the procedure described by Sharpless [46] (Scheme 1 and Table 1). The click chemistry process, carried out with equimolar amounts of the respective dipolarophiles, afforded in all the cases the corresponding C-5’-triazolyl-2’-oxa-3’-aza-4’a-carbanucleosides **13** and **14** in good yields (79–89%). According to other copper-catalyzed azide–alkyne cycloadditions, no traces of 1,5-regioisomers were observed [47–48].

The structure of the obtained compounds was assessed according to ^1^H NMR, ^13^C NMR and MS data. In particular, the ^1^H NMR spectra of 5-methyl-1-[(3*RS,*5*SR*)-2-methyl-3-(1*H*-1,2,3-triazol-1-ylmethyl)isoxazolidin-5-yl]pyrimidine-2,4(1*H,*3*H*)diones **13** and 5-methyl-1-[(3*RS,*5*RS*)-2-methyl-3-(1*H*-1,2,3-triazol-1-ylmethyl)isoxazolidin-5-yl]pyrimidine-2,4(1*H,*3*H*)diones **14** show, besides the resonances of the protons of the isoxazolidine unit, diagnostic resonances at 7.25–7.75 ppm, as a singlet, for the proton of the triazole system, and at 4.50–5.10 and 4.25–4.75 ppm, respectively in **13** and **14**, as a doublet of doublets, for the methylene group at C-4’ position.

### Biological tests

The antiproliferative effect of the obtained derivatives was tested on a panel of cell lines: african green monkey kidney cells (Vero and BS-C-1), human epidermoid carcinoma larynx cells (HEp-2), Madin–Darby canine kidney (MDCK), and human foreskin fibroblast cells (HFF). In these assays the cells were in the logarithmic phase of growth.

Inhibition of cell proliferation, with a CC_50_ ranging from 5 to 40 µM (Table 2), has been observed for all the new synthesized compounds. In particular, compound **14d** showed a high level of inhibitory activity with CC_50_ values of 5 μM for all the utilized cell lines, while compounds **13c**, **13e**, **13d**, **14c**, **14e**, **14f** and **14g** show the same CC_50_ values only for HFF cells.

**Table 2:** Biological activity of C-5’-triazolyl-2’-oxa-3’-aza-4’a-carbanucleosides **13a–g** and **14a–g**.

	CC_50_ μM^a^				
Compound	VERO	HEp2	MDCK	HFF	BS-C-1

**13a**	10	40	10	10	10
**14a**	20	40	20	20	20
**13b**	40	40	30	30	30
**14b**	20	40	20	20	10
**13c**	20	40	20	5	20
**14c**	20	40	20	5	20
**13d**	20	20	20	5	20
**14d**	5	5	5	5	5
**13e**	20	40	20	5	20
**14e**	20	40	20	5	20
**13f**	10	40	10	40	10
**14f**	20	40	20	5	20
**13g**	10	20	10	5	10
**14g**	10	20	10	5	10

^a^CC_50_: Concentration which inhibited cell growth by 50% as compared with control cultures. Values are mean ± 0.5 S.D. (estimated maximal standard deviation) of three separate assays.

Noteworthy, the relative *cis, trans* configuration of **13** and **14** does not seem to affect the biological effect. The cytostatic activity of the compounds was particularly exploited against HFF cell proliferation.

According to our initial hypothesis, the presence of the triazole linker at C-5’ position in the 2’-oxa-3’-aza-4’a-carbanucleoside skeleton induces a different biological effect with respect to 2’-oxa-3’-aza-4’a-carbanucleosides devoid of the triazole unit, such as compounds **2** and **8**, which are endowed with antiviral activity, but do not show any cytotoxicity

The ability of compounds **13a–g** and **14a–g** to interfere with the replication of different DNA and RNA viruses was also evaluated, by using the subsequent cell-virus tests: (a) Vero cell for poliovirus 1, human echovirus 9**,** herpes simplex type 1 (HSV-1); (b) HEp-2 cell for Coxsackievirus B1, adenovirus type 2; (c) human foreskin fibroblast cells (HFF) for cytomegalovirus (CMV); (d) BS-C-1 cell (African green monkey kidney) for varicella-zoster virus (VZV); (e) Madin–Darby canine kidney (MDCK) for influenza virus A/Puerto Rico/8/34 H1N1 (PR8). Acyclovir was used as the reference compound. For the synthesized compounds, no inhibitory activity against any virus was detected until 250 μM.

### Biological assays

**Cells.** Biological assays have been performed on African green monkey kidney cells (Vero and BS-C-1), human epithelial type 2 cells (HEp-2), human foreskin fibroblast cells (HFF), Madin-Darby canine kidney (MDCK). All cell lines were obtained from the American Type Culture Collection. The cell cultures were maintained at 37 °C in a humidified atmosphere with 5% CO_2_ and grown in D-MEM (Dulbecco's modified Eagle’s Minimum Essential medium) supplemented with 10% FCS (fetal calf serum, 2 mM/L glutamine, 0.1% sodium bicarbonate, 200 μg/mL of streptomycin and 200 units/mL of penicillin G. The maintenance medium (DMEM with 2% heat inactivated FCS) was used to culture the viruses.

**Cell viability.** The cytotoxicity of the tested compounds was evaluated by measuring the effect created on cell morphology and/or cell growth (cytostatic activity). Cell monolayers were prepared in 24-well tissue culture plates and exposed to various concentrations of the compounds. Cytotoxicity was recorded as morphological variations (such as rounding up, shrinking and detachment) at 24, 48, 72 and 96 h, using light microscopy. Cytotoxicity was expressed as the minimum cytotoxic concentration (MCC) that caused a microscopically detectable variation of cell morphology. The extent of cytostatic activity was measured as inhibition of cell growth using the MTT method, as previously described [49–50]. The 50% cytotoxic dose (CC_50_) is the compound concentration required to reduce cell proliferation by 50% relative to the absorbance of the untreated control. CC_50_ values were estimated from graphic plots of the percentage of control as a function of the concentration of the test compounds.

**Test compounds**. Compounds **13** and **14** were dissolved in DMSO and diluted in maintenance medium to achieve the final required concentration. The final dilution of test compounds contained a maximum concentration of 0.01% DMSO, which had no effect on the viability of the cell lines. Stock solutions of acycloguanosine (Sigma, USA) were prepared in distilled water, filtered through 0. 2 μm filter and stored at 4 °C until use.

**Viruses**. In the antiviral assays the following viruses were used: Poliovirus 1 (Sabin strain: VR-1562), Human echovirus 9 (VR-1050), Herpes simplex type 1 (HSV-1: VR-260), Coxsackievirus B1 (VR-28), adenovirus type 2 (VR-1080), Cytomegalovirus (CMV: VR-538), varicella-zoster virus (VZV: VR-1367), influenza virus A/Puerto Rico/8/34 H1N1 (PR8). Viruses were obtained from the American Type Culture Collection. The tests on the antiviral activity were carried out by the 50% plaque reduction assay or by 50% virus-induced cytopathogenicity, as previously described [51]. The concentration of the compound that inhibit the formation of viral plaques or virus-induced cytopathogenicity by 50% is expressed as EC50.

## Conclusion

In summary, starting from 5’-azido-2’-oxa-3’-azanucleosides, a new series of C-5’-triazolyl-2’-oxo-3’-aza-4’a-carbanucleosides has been synthesized by using a CuI-catalyzed Huisgen [3 + 2] cycloaddition with substituted alkynes. The biological assays indicate that these compounds inhibit the cell proliferation of Vero, BS-C-1, HEp-2, MDCK, and HFF cells by 50% (CC_50_) at concentrations in the range of 5.0–40.0 μM. No antiviral activity at subtoxic concentrations was observed.

## Supporting Information

File 1 Preparation and analytical data of compounds **9**–**14**. Copies of ^1^H and ^13^C NMR spectra of all new compounds.

## References

[1] Mehellou Y, De Clercq E (2010). J Med Chem.

[2] Štambaský J, Hocek M, Kočovský P (2009). Chem Rev.

[3] Galmarini C M, Popowycz F, Joseph B (2008). Curr Med Chem.

[4] Balestrieri E, Matteucci C, Ascolani A, Piperno A, Romeo R, Romeo G, Chiacchio U, Mastino A, Macchi B (2008). Antimicrob Agents Chemother.

[5] De Clercq E (2004). Nat Rev Microbiol.

[6] Galmarini C M, Mackey J R, Dumontet C (2002). Lancet Oncol.

[7] Pathak T (2002). Chem Rev.

[8] Ferrero M, Gotor V (2000). Chem Rev.

[9] Saag M S (2012). Top Antivir Med.

[10] Bonate P L, Arthaud L, Cantrell W R, Stephenson K, Secrist J A, Weitman S (2006). Nat Rev Drug Discovery.

[11] Hatse S, De Clercq E, Balzarini J (1999). Biochem Pharmacol.

[12] Lauria F, Benfenati D, Raspadori D, Rondelli D, Zinzani P L, Tura S (1993). Leuk Lymphoma.

[13] Romeo G, Chiacchio U, Corsaro A, Merino P (2010). Chem Rev.

[14] Merino P (2006). Curr Med Chem.

[15] Hirota K, Monguchi Y, Sajiki H, Chu C K (2002). Synthesis of Purine Acyclonucleosides via Ribofuranose-Ring Cleavage of Purine Nucleosides by Diisobutylaluminum Hydride. Recent Advances in Nucleosides: Chemistry and Chemotherapy.

[16] Littler E, Zhou E E, Taylor J B, Triggle D J (2006). Comprehensive Medicinal Chemistry II.

[17] Sharma P L, Nurpeisov V, Hernandez-Santiago B, Beltran T, Schinazi R F (2004). Curr Top Med Chem.

[18] Bortolini O, Mulani I, De Nino A, Maiuolo L, Nardi M, Russo B, Avnet S (2011). Tetrahedron.

[19] Piperno A, Chiacchio M A, Iannazzo D, Romeo R (2006). Curr Med Chem.

[20] Maiuolo L, Bortolini O, De Nino A, Russo B, Gavioli R, Sforza F (2014). Aust J Chem.

[21] Merino P, Tejero T, Unzurrunzaga F J, Franco S, Chiacchio U, Saita M G, Iannazzo D, Piperno A, Romeo G (2005). Tetrahedron: Asymmetry.

[22] Chiacchio U, Genovese F, Iannazzo D, Librando V, Merino P, Rescifina A, Romeo R, Procopio A, Romeo G (2004). Tetrahedron.

[23] Chiacchio U, Corsaro A, Pistarà V, Rescifina A, Iannazzo D, Piperno A, Romeo G, Romeo R, Grassi G (2002). Eur J Org Chem.

[24] Chiacchio U, Corsaro A, Iannazzo D, Piperno A, Procopio A, Rescifina A, Romeo G, Romeo R (2001). Eur J Org Chem.

[25] Romeo R, Carnovale C, Giofrè S V, Monciino G, Chiacchio M A, Sanfilippo C, Macchi B (2014). Molecules.

[26] Romeo R, Navarra M, Giofrè S V, Carnovale C, Cirmi S, Lanza G, Chiacchio M A (2014). Bioorg Med Chem.

[27] Romeo R, Giofrè S V, Garozzo A, Bisignano B, Corsaro A, Chiacchio M A (2013). Bioorg Med Chem.

[28] Romeo R, Carnovale C, Giofrè S V, Romeo G, Macchi B, Frezza C, Marino-Merlo F, Pistarà V, Chiacchio U (2012). Bioorg Med Chem.

[29] Piperno A, Giofrè S V, Iannazzo D, Romeo R, Romeo G, Chiacchio U, Rescifina A, Piotrowska D G (2010). J Org Chem.

[30] Chiacchio U, Borrello L, Iannazzo D, Merino P, Piperno A, Rescifina A, Richichi B, Romeo G (2003). Tetrahedron: Asymmetry.

[31] Chiacchio U, Corsaro A, Iannazzo D, Piperno A, Rescifina A, Romeo R, Romeo G (2001). Tetrahedron Lett.

[32] Romeo R, Giofrè S V, Iaria D, Sciortino M T, Ronsisvalle S, Chiacchio M A, Scala A (2011). Eur J Org Chem.

[33] Singhal N, Sharma P K, Kumar N, Duhe R (2011). Chem Biol Interface.

[34] Singh R J, Singh D K (2009). E-J Chem.

[35] Moorhouse A D, Moses J E (2008). ChemMedChem.

[36] Lutz J-F (2007). Angew Chem, Int Ed.

[37] Angell Y L, Burgess K (2007). Chem Soc Rev.

[38] Tome A C, Stor R, Gilchrist T (2004). Product class 13: 1,2,3-triazoles.

[39] Rowan A S, Nicely N I, Cochrane N, Wlassoff W A, Claiborne A, Hamilton C J (2009). Org Biomol Chem.

[40] Romeo R, Giofrè S V, Carnovale C, Campisi A, Parenti R, Bandini L, Chiacchio M A (2013). Bioorg Med Chem.

[41] Romeo R, Giofrè S V, Carnovale C, Chiacchio M A, Campisi A, Mancuso R, Cirmi S, Navarra A (2014). Eur J Org Chem.

[42] Carnovale C, Iannazzo D, Nicolosi G, Piperno A, Sanfilippo C (2009). Tetrahedron: Asymmetry.

[43] Chiacchio U, Rescifina A, Iannazzo D, Piperno A, Romeo R, Borrello L, Sciortino M T, Balestrieri E, Macchi B, Mastino A (2007). J Med Chem.

[44] Iannazzo D, Piperno A, Pistarà V, Rescifina A, Romeo R (2002). Tetrahedron: Asymmetry.

[45] Ward D I, Jeffs S M, Coe P L, Walker R T (1993). Tetrahedron Lett.

[46] Kolb H C, Finn M C, Sharpless K B (2001). Angew Chem, Int Ed.

[47] Spiteri C, Moses J E (2010). Angew Chem, Int Ed.

[48] Tornøe C W, Christensen C, Meldal M (2002). J Org Chem.

[49] Denizot F, Lang R (1986). J Immunol Methods.

[50] Cutrì C C C, Garozzo A, Siracusa M A, Sarvà M C, Tempera G, Geremia E, Pinizzotto M R, Guerrera F (1998). Bioorg Med Chem.

[51] Garozzo A, Cutrì C C C, Castro A, Tempera G, Guerrera F, Sarvà M C, Geremia E (2000). Antiviral Res.

