# Supplementary material for: C-5’-Triazolyl-2’-oxa-3’-aza-4’a-carbanucleosides: Synthesis and biological evaluation
Source: Beilstein J Org Chem. 2015 Mar 9;11:328–34. doi: 10.3762/bjoc.11.38 (PMC4362054; doi:10.3762/bjoc.11.38)

Supporting Information

for

C-5’-Triazolyl-2’-oxa-3’-aza-4’a-carbanucleosides: Synthesis and biological evaluation

Roberto Romeo*[§1], Caterina Carnovale[1], Salvatore V. Giofrè*[¶1], Maria A. Chiacchio[2], Adriana Garozzo[3], Emanuele Amata[2], Giovanni Romeo[1] and Ugo Chiacchio[2]

Address: [1]Dipartimento Scienze del Farmaco e dei Prodotti per la Salute, University of Messina, Via S.S. Annunziata, 98168 Messina, Italy, [2]Dipartimento di Scienze del Farmaco, University of Catania, Via A. Doria 6, 95125-Catania, Italy and [3]Dipartimento di Scienze Bio-Mediche, University of Catania,Via Androne 81, 95124 Catania, Italy

Email: Roberto Romeo - robromeo@unime.it; Salvatore V. Giofrè - sgiofre@unime.it

*Corresponding author

[§]Tel.: +39-090-356230; fax: +39-090-6766474

[¶]Tel.: +39-090-6766566; fax: +39-090-6766474

Table of contents

Experimental section……………………………………………………………S2

^{1}H and ^{13}C NMR ……………………………………………………………….S7

Experimental section

General: Solvents and reagents are commercial. HRMS were determined with a SQ Quantum XLS Triple Quadrupole GC-MS/MS. NMR spectra (^{1}H NMR at 300 and 500 MHz, ^{13}C NMR at 75 and 126 MHz) were recorded with Varian instruments and are reported in ppm relative to TMS. Merck silica gel 60-F254 precoated aluminum plates have been used for thin-layer chromatographic separations. Flash chromatography was performed on Merck silica gel (200–400 mesh). Preparative separations were carried out by a MPLC Büchi C-601 by using Merck silica gel 0.040–0.063 mm. Compound **8** was synthesized according to literature procedures.[11]

Synthesis of ((3*RS*,5*SR*)-5-(3,4-dihydro-5-methyl-2,4-dioxopyrimidin-1(2*H*)-yl)-2-methylisoxazolidin-3-yl)methyl 4-methylbenzensulfonate (9).

To a solution of compound **8** (0.386 g, 1.6 mmol) in CH_2Cl_2 (20 mL), Et_3N (0.25 mL, 1.8 mmol) was added. TsCl (0.349 g, 1.8 mmol) was then added slowly at 0°C and the reaction mixture was left under stirring at room temperature for 24 h. After removal of the solvent under vacuum, the residue was purified by MPLC, using CH_2Cl_2/MeOH 99:1, to give **9** in 70% yield (0.443 g).

White solid, m.p. 134-136 °C. ^{1}H NMR (500 MHz, $CDCl_3$): □ δ = 1.77 (s,3H); 1.93-2.04 (m, 1H), 2.32 (s, 3H); 2.63 (s, 3H); 2.82-2.89 (m, 2H); 3.89-4.07 (m, 2H), 5.94- 6.02 (m, 1H), 7.23 (d, *J* = 8.2 Hz, 2H), 7.46 (s, 1H); 7.63 (d, *J* = 8.2 Hz, 2H), 9.37 (bs, 1H). ^{13}C NMR (126 MHz, $CDCl_3$): δ = 8.54, 17.48, 36.53, 40.26, 61.81, 63.64, 89.98, 106.58, 123.67, 125.90, 131.73, 138.66, 144.09, 151.95, 160.09. HRMS: calcd for $C_{17}H_{21}N_3O_6SNa^+$ 418.1043, found 418.1050.

Synthesis of (3*RS*,5*SR*)-1-(3-(iodomethyl)-2-methylisoxazolidin-5-yl)-5-methylpyrimidine-2,4-(1*H*,3*H*) dione (10)

Compound **9** (0.32 g, 0.81 mmol) was added to a solution of NaI (0.600 g, 4 mmol) in acetone (20 mL) and the reaction mixture was refluxed for 72 h**.** After cooling, acetone was removed under vacuum and chloroform was added to the residue. The resulting organic phase was filtered and evaporated. The residue was purified by MPLC, using CH_2Cl_2/MeOH 99 :1, to give the compound **10** in 93% yield (0.260 g).

Pale yellow solid, m.p. 157-159 °C. ^{1}H NMR (300 MHz, $CDCl_3$): δ = 1.96 (s, 3H), 2.20 (ddd, *J* = 13.7, 9.3, 4.1 Hz, 1H), 2.51-2.64 (m, 1H), 2.76 (s, 3H), 3.05-3.18 (m, 2H), 3.27 (dd, *J* = 10.7, 3.3 Hz, 1H), 6.16 (dd, *J* = 7.8, 4.1 Hz, 1H), 7.77 (s, 1H), 8.54 (bs, 1H). ^{13}C NMR (126 MHz, $CDCl_3$): δ = 8.44; 25.45, 38.99, 41.18, 63.28, 77.44, 110.20, 132.30, 150.20, 163.4. HRMS: calcd for $C_{10}H_{14}N_3O_3INa^+$ 373.9972, found 373.9981.

Synthesis of (3*RS*,5*SR*)-1-(3-(azidomethyl)-2-methylisoxazolidin-5-yl)-5-methylpyrimidine-2,4-(1*H*,3*H*)-dione (11) and (3*RS*,5*RS*)-1-(3-(azidomethyl)-2-methylisoxazolidin-5-yl)-5-methyl-pyrimidine-2,4-(1*H*,3*H*)-dione (12)

To a solution of compound **10** (0.197 g, 0.56 mmol) in acetonitrile (4 mL) and H_2O (8 mL), NaN_3 (0.04 g, 0.61 mmol) and NH_4Cl (0.06 g, 1. 12 mmol) were added. The resulting mixture was stirred at 50 °C for 48 h. The solvent was evaporated

under reduced pressure and the residue was washed with $CHCl_3$ (10 mL). After filtration, the residue was purified by silica gel column chromatography (CH_2Cl_2/MeOH= 99:1).

The first eluted product was the *cis* derivative **11**. Yield 57%, yellow oil. ^{1}H NMR (300 MHz, $CDCl_3$): δ = 1.49-1.66 (m, 1H); 1.93 (s, 3H), 2.26 (dd, 11.7, 5.1 Hz, 1H), 2.34-2.42 (m, 1H), 2.70 (s, 3H), 3.17 (dd, *J* = 11.7, 4.7 Hz, 1H), 3.85-4.00 (m, 1H), 5.99 (dd, *J* = 11.7, 5.1 Hz, 1H), 7.14 (s, 1H), 8.96 (bs, 1H). ^{13}C NMR (126 MHz, $CDCl_3$): δ = 16.20, 42.02; 43.40, 51.02, 65.60, 89.00, 111.20, 138.40, 152.60, 164.20. The second eluted product was the *trans* derivative **12**. Yield 28%, yellow oil. ^{1}H NMR (500MHz, $CDCl_3$): δ = 1.95 (s,3H) ; 2.18 (ddd, *J* = 13.2, 9.2, 3.7 Hz, 1H); 2.83 (s, 3H); 2.91-3.01 (m,1H); 3.05-3.09 (m, 1H); 4.05 (dd, *J* = 11.9, 5.9 Hz, 1H); 4.31 (dd, *J* = 11.9, 3.8 Hz, 1H); 6.14 (dd, *J* = 7.7, 3.7 Hz, 1H); 7.67 (s,1H); 8.38 (bs, 1H). ^{13}C NMR (126 MHz, $CDCl_3$): δ = 20.66; 41.52; 44.38; 62.63; 66.29; 82.45; 110.55; 135.92; 150.23; 163.44.

General procedure for the synthesis of 5'-[1,2,3]-triazolyl-2'-oxa-3'-azanucleosides 13 and 14.

To a solution of azide **11** or **12** (0.4 mmol) in *tert*-butanol:water (1:1, 20 mL) mixture, NEt_3 (0.4 mmol), the corresponding alkyne **17** (0.4 mmol), $CuSO_4.5H_2O$ (0.1 mmol) and sodium ascorbate (0.2 mmol) were added. The reaction mixture was stirred at room temperature for 5 h. After the completion of reaction, the solvent was evaporated under reduced pressure and the residue was extracted with dichloromethane (20 mL x 3). The organic phase was dried over sodium sulfate, filtered and evaporated under reduced pressure. The residue was purified by MPLC, using CH_2Cl_2: MeOH 99:1, to give the corresponding triazolyl-2'oxa-3'azanucleosides.

1-((3*RS*,5*SR*)-3-((4-(2-hydroxyethyl)-1*H*-1,2,3-triazol-1-yl)methyl)-2-methylisoxazolidin-5-yl)-5-methylpyrimidine-2,4(1*H*,3*H*)-dione (13a). Yield 88%, white solid, m.p. 215-218 °C. ^{1}H NMR (500 MHz, DMSO): δ = 1.78 (s, 3H), 2.25-2.29 (m, 1H), 2.43-2.47 (m, 1H), 2.64 (s, 3H), 2.71-2.74 (m, 3H), 3.44 (dd , *J* = 11.7, 4.2 Hz, 1H); 3.62 (dt, *J* = 6.9, 5.3 Hz, 2H); 4.69 (t, *J*= 5.3 Hz, 1H); 5.03-5.06 (m, 1H); 5.97 (dd, *J* = 11.3, 2.4 Hz, 1H); 7.71 (s, 1H); 8.01 (s, 1H). ^{13}C NMR (126 MHz, DMSO): δ = 16.77, 34.24, 38.40, 50.62, 59.71, 65.28, 65.59, 84.55, 114.65, 126.32, 141.22, 149.61, 155.30, 168.73. IR (nujol): v_{max} 3450, 1698. HRMS: calcd for $C_{14}H_{20}N_6O_4Na^+$ 359.1438, found 359.1442.

1-((3*RS*,5*SR*)-3-((4-(hydroxymethyl)-1*H*-1,2,3-triazol-1-yl)methyl)-2-methylisoxazolidin-5-yl)-5-methylpyrimidine-2,4(1*H*,3*H*)-dione (13b). Yield 84%, white solid, m.p 205-210 °C. ^{1}H NMR (500 MHz, DMSO): δ = 1.78 (s, 3H); 2.24-2.30 (m, 1H), 2.50-2.54 (m, 1H), 2.64 (s, 3H), 2.75 (dd, *J* = 11.2, 9.8 Hz, 1H), 3.41-3.49 (m, 1H), 4.52 (d, *J* = 5.5Hz, 1H), 5.07-5.13 (m, 1H), 5.21 (t, *J* = 5.5 Hz, 1H), 5.98 (dd, *J* = 11.2, 2.1 Hz, 1H), 7.72 (s, 1H), 8.13 (s, 1H), 9.40 (bs, 1H). ^{13}C NMR (126 MHz, DMSO): δ = 12.36, 33.70, 46.23, 55.33, 60.89, 61.85, 79.85, 110.27, 121.93, 136.59; 148.59, 150.32, 163.78. IR (nujol): v_{max} 3450, 1696. HRMS: calcd for $C_{13}H_{18}N_6O_4Na^+$ 345.1282, found 345.1286.

5-methyl-1-((3*RS*,5*SR*)-2-methyl-3-((4-propyl-1*H*-1,2,3-triazol-1-yl)methyl)isoxazolidin-5-yl)pyrimidine-2,4(1*H*,3*H*)-dione (13c). Yield 80%, white solid, .m.p. 132-137 °C. ^{1}H NMR (500 MHz, $CDCl_3$): δ = 0.96 (t, *J* = 7.4 Hz, 3H), 1.64-1.72 (m,

2H), 1.94 (s, 3H), 2.22-2.32 (m, 1H), 2.39-2.49 (m, 1H), 2.68 (t, *J* = 7.6 Hz, 2H), 2.76 (s, 3H), 3.05 (dd, *J* = 11.7, 11.2 Hz, 1H), 3.38 (dd, *J* = 11.7,4.0 Hz,1H), 4.91-4.97 (m, 1H), 6.15 (dd, *J* = 11.0, 2.5 Hz, 1H), 7.22 (s, 1H); 7.34 (s, 1H), 9.13 (bs, 1H). ^{13}C NMR (126 MHz, $CDCl_3$): *δ* = 7.74, 8.98, 17.88, 22.83, 30.52, 41.48, 50.55, 56.43, 75.36, 106.95, 115.19, 130.18, 143.49, 145.21, 158.59. IR (nujol): ν_{max} 1699. HRMS: calcd for $C_{15}H_{22}N_6O_3Na^+$ 357.1646, found 357.1642.

5-methyl-1-((3*RS*,5*SR*)-2-methyl-3-((4-phenyl-1*H*-1,2,3-triazol-1-yl)methyl)isoxazolidin-5-yl)-pyrimidine-2,4(1*H,*3*H*)-dione (13d). Yield 78%, white solid, m.p. 268-272 °C. ^{1}H NMR (300 MHz, $CDCl_3$): *δ* = 1.96 (s, 3H), 2.34-2.60 (m, 2H), 2.80 (s, 3H), 3.12-3.16 (m, 1H), 3.39-3.53 (m, 1H), 4.96-5.13 (m, 1H), 6.18 (dd, *J* = 10.9, 2.8 Hz, 1H), 7.20-7.25 (m, 2H), 7.32-7.51 (m, 3H), 7.81 (s, 1H), 7.84 (s, 1H), 8.12 (bs, 1H). ^{13}C NMR (126 MHz, $CDCl_3$): *δ* = 12.40, 30.70, 46.09, 55.20, 61.27, 80.01, 118.80, 125.58, 128.25, 128.71, 130.25, 134.83, 138.20, 146.32, 150.41, 163.72. IR (nujol): ν_{max} 1697. HRMS: calcd for $C_{18}H_{20}N_6O_3Na^+$ 391.1489, found 391.1493.

1-((3*RS*,5*SR*)-3-((4-(4-methoxyphenyl)-1*H*-1,2,3-triazol-1-yl)methyl)-2-methylisoxazolidin-5-yl)-5-methylpyrimidine-2,4(1*H,*3*H*)-dione (13e): Yield 78%, white solid, m.p. 215-218 °C. ^{1}H NMR (500 MHz, DMSO): *δ* = 1.51 (s, 3H), 2.14-2.25 (m, 1H), 2.68 (s, 3H), 2.95-2.99 (m, 1H), 3.12-3.14 (m, 1H), 3.75 (s, 3H), 4.61-4.70 (m, 2H), 6.05 (dd, *J* = 8.0, 4.0 Hz, 1H), 6.97 (d, *J* = 8.6 Hz, 2H), 7.14 (s, 1H), 7.73 (d, *J* = 8.6 Hz, 2H), 8.43 (s, 1H). ^{13}C NMR (126 MHz, DMSO): *δ* = 10.43, 45.50, 50.18, 55.50, 60.30, 65.40, 82.41, 109.67, 114.64, 122.34, 128.50, 132.01, 135.90, 147.70, 152.44, 158.01, 160.65. IR (nujol): ν_{max} 1697. HRMS: calcd for $C_{19}H_{22}N_6O_4Na^+$ 421.1595, found 421.1593.

1-((3*RS*,5*SR*)-3-((4-(4-fluorophenyl)-1*H*-1,2,3-triazol-1-yl)methyl)-2-methylisoxazolidin-5-yl)-5-methylpyrimidine-2,4(1*H,*3*H*)-dione (13f). Yield 85%, white solid, m.p. 269-271 °C. ^{1}H NMR (500 MHz, $CDCl_3$): *δ* = 1.97 (s, 3H), 2.32-2.42 (m, 1H), 2.48-2.57 (m, 1H), 2.80 (s, 3H), 3.12 (dd, *J* = 11.3, 9.9 Hz, 1H), 3.43-3.53 (m, 1H), 4.95-5.09 (m, 1H), 6.17 (dd, *J*=11.2, 2.5 Hz, 1H), 7.13 (t, *J* = 8.7Hz, 2H), 7.23 (s, 1H), 7.76 (s, 1H), 7.77-7.85 (m, 2H), 8.00 (bs, 1H). ^{13}C NMR (126 MHz, DMSO): *δ* = 17.09, 38.36, 51.02, 60.25, 65.49, 84.61, 115.03, 121.06 (d, *J* = 21.6 Hz), 125.45, 132.28, 141.26, 150.49, 155.33, 166.96 (d, *J* = 244.4 Hz), 168.66. IR (nujol): ν_{max} 1685. HRMS: calcd for $C_{18}H_{19}N_6O_3FNa^+$ 409.1395, found 409.1399.

1-((3*RS*,5*SR*)-2-methyl-3-((4-(4-pentylphenyl)-*1H*-1,2,3-triazol-1-yl)methyl)isoxazolidin-5-yl)-5-methylpyrimidine-2,4(*1H,3H*)-dione (13g). Yield 89%, white solid, m.p. 259-260 °C. ^{1}H NMR (300 MHz, $CDCl_3$): *δ* = 0.77 (t, *J* = 6.6Hz, 3H), 1.29-1.40 (m, 4H), 1.55-1.71 (m, 2H), 1.95 (s, 3H); 2.27-2.55 (m, 2H), 2.62 (t, *J* = 7.4, 2H), 2.78 (s, 3H), 3.04-3.19 (m, 1H), 3.38-3.51 (m, 1H), 4.95-5.12 (m, 1H), 6.18 (dd, *J* = 11.0, 2.6 Hz, 1H), 7.26 (d, *J* = 8.1, 2H), 7.28 (s, 1H), 7.72 (d, *J* = 8.1 Hz, 2H), 7.78 (s, 1H), 8.71 (s, 1H). ^{13}C NMR (126 MHz, DMSO): *δ* = 11.88, 13.86, 21.71, 30.54, 31.75, 31.90, 33.37, 46.13, 55.10, 60.57, 79.66, 110.05, 120.07, 121.30, 125.28, 128.74, 136.33, 146.50, 149.01, 168.20. IR (nujol): ν_{max} 1695. HRMS: calcd for $C_{23}H_{30}N_6O_3Na^+$ 461.2272, found 461.2277.

1-((3*RS*,5*RS*)-3-((4-(2-hydroxyethyl)-1*H*-1,2,3-triazol-1-yl)methyl)-2-methylisoxazolidin-5-yl)-5-methylpyrimidine-2,4(1*H,*3*H*)-dione (14a). Yield 79%, white solid.m.p. 202-206 °C; ^{1}H NMR (500 MHz, $CDCl_3$): δ = 1.93 (s,3H); 2.17-2.23 (m, 1H), 2.73 (s, 3H), 2.94 (t, *J* = 5.8 Hz, 2H), 3.01-3.12 (m, 1H), 3.20-3.30 (m, 1H), 3.89-3.99 (m, 2H); 4.44 (dd, *J* = 14.4, 4.9 Hz, 1H), 4.57 (dd, *J* = 14.4, 5.3 Hz, 1H), 6.10 (dd, *J* = 7.6, 3.9 Hz, 1H), 7.32 (s, 1H), 7.45 (s, 1H), 8.03 (bs, 1H). ^{13}C NMR (126 MHz, $CDCl_3$): δ = 14.75, 32.44, 39.41, 51.01, 58.99, 64.38, 65.02, 84.65, 114.01, 125.92, 142.34, 150.40, 157.60, 160.01. IR (nujol): ν_{max} 3450, 1695. HRMS: calcd for $C_{14}H_{20}N_6O_4Na^+$ 359.1438, found 359.1443.

1-((3*RS*,5*RS*)-3-((4-(hydroxymethyl)-1*H*-1,2,3-triazol-1-yl)methyl)-2-methylisoxazolidin-5-yl)-5-methylpyrimidine-2,4(1*H,*3*H*)-dione (14b). Yield 81%, white solid.m.p. 198-201 °C, ^{1}H NMR (500 MHz, $CDCl_3$): δ = 1.92 (s, 3H), 2.17-2.23 (m, 1H), 2.74 (s, 3H), 3.01-3.12 (m, 1H), 3.19-3.31 (m, 1H), 3.98 (dd, *J* = 7.6, 3.9 Hz, 2H), 4.45 (dd, *J* = 14.5, 4.8 Hz, 1H), 4.60 (dd, *J* = 14.5, 5.2 Hz, 1H), 6.08 (dd, *J* = 7.6, 3.9 Hz, 1H), 7.30 (s, 1H), 7.57 (s, 1H), 8.11 (bs, 1H). ^{13}C NMR (126 MHz, $CDCl_3$): δ = 13.93, 38.52, 41.29, 43.63, 56.71, 66.94, 82.35, 110.09, 120.82, 122.73, 134.93, 157.60, 161.04. IR (nujol): ν_{max} 3496, 1697. HRMS: calcd for $C_{13}H_{18}N_6O_4Na^+$ 345.1282, found 345.1287.

5-methyl-1-((3*RS*,5*RS*)-2-methyl-3-((4-propyl-1*H*-1,2,3-triazol-1-yl)methyl)isoxazolidin-5-yl)pyrimidine-2,4(1*H,*3*H*)-dione (14c). Yield 83%, white solid, m.p. 168-170 °C, ^{1}H NMR (300 MHz, $CDCl_3$): δ = 0.94 (t, *J* = 7.3 Hz, 3H), 1.66 (sextet, *J* = 7.3, 2H), 1.90 (s, 3H), 2.08-2.22 (m, 1H), 2.60-2.68 (m, 2H), 2.70 (s, 3H), 3.04 (dt, *J* = 14.4, 7.3 Hz, 1H), 3.15-3.29 (m, 1H), 4.42 (dd, *J* = 14.4, 4.8 Hz, 1H), 4.55 (dd, *J* = 14.4, 5.4 Hz, 1H), 6.10 (dd, *J* = 7.6, 4.0 Hz, 1H), 7.31 (s, 1H), 7.32 (s, 1H), 9.02 (bs, 1H). ^{13}C NMR (126 MHz, $CDCl_3$): δ = 12.64, 13.71, 22.63, 27.55, 41.69, 43.87, 49.93, 67.03, 82.34, 110.73, 121.69, 136.26, 148.64, 150.37, 167.73. IR (nujol): ν_{max} 1698. HRMS: calcd for $C_{15}H_{22}N_6O_3Na^+$ 357.1646, found 357.1648.

5-methyl-1-((3*RS*,5*RS*)-2-methyl-3-((4-phenyl-1*H*-1,2,3-triazol-1-yl)methyl)isoxazolidin-5-yl)pyrimidine-2,4(1*H,*3*H*)-dione (14d). Yield 82%, white solid, m.p. 233-235 °C, ^{1}H NMR (300 MHz, $CDCl_3$): δ = 1.72 (s, 3H), 2.17-2.30 (m, 1H), 2.78 (s, 3H), 3.03-3.19 (m, 1H), 3.25-3.33 (m, 1H), 4.45 (dd, *J* = 14.4, 4.3 Hz, 1H), 4.58 (dd, *J* = 14.4, 5.1 Hz, 1H), 6.13 (dd, *J* = 7.7, 4.1 Hz, 1H), 7.21 (s,1H), 7.33-7.39 (m, 3H), 7.41-7.46 (m, 2H), 7.82(s, 1H), 7.90 (bs, 1H). ^{13}C NMR (126 MHz, $CDCl_3$): δ = 12.38, 29.67, 41.23, 43.84, 49.67, 66.90, 82.04, 110.52, 111.49, 120.30, 125.65, 128.35, 128.96, 134.89, 151.04, 163.19. IR (nujol): ν_{max} 1699. HRMS: calcd for $C_{18}H_{20}N_6O_3Na^+$ 391.1489, found 391.1491.

1-((3*RS*,5*RS*)-3-((4-(4-methoxyphenyl)-1*H*-1,2,3-triazol-1-yl)methyl)-2-methylisoxazolidin-5-yl)-5-methylpyrimidine-2,4(1*H,*3*H*)-dione (14e). Yield 82%, white solid, m.p. 202-205 °C, ^{1}H NMR (300 MHz, $CDCl_3$): δ = 1.74 (s, 3H), 2.13-2.24 (m, 1H), 2.70 (s, 3H), 3.05-3.15 (m, 1H), 3.25-3.30 (m, 1H), 3.85 (s, 3H), 4.49 (dd, *J* = 13.7, 4.7 Hz, 1H), 4.65 (dd, *J* = 13.7, 4.1 Hz, 1H), 6.11-6.20 (m, 1H), 6.97 (d, *J* = 8.7 Hz, 2H), 7.22 (s, 1H), 7.72-7.76 (m, 3H), 7.90 (bs, 1H). ^{13}C NMR (126 MHz, DMSO): δ = 12.57, 43.58,

49.98, 55.49, 59.15, 66.55, 81.38, 110.21, 114.70, 121.97, 126.88, 130.15,136.98, 146.71, 150.43, 159.45, 163.99. IR (nujol): v_{max} 1696. HRMS: calcd for $C_{19}H_{22}N_6O_4Na^+$ 421.1595, found 421.1598.

1-((3*RS*,5*RS*)-3-((4-(4-fluorophenyl)-1*H*-1,2,3-triazol-1-yl)methyl)-2-methylisoxazolidin-5-yl)-5-methylpyrimidine-2,4(1*H*,3*H*)-dione (14f). Yield 84%, white solid, m.p. 230-233 °C, ^{1}H NMR (500 MHz, DMSO): δ = 1.50 (s, 3H), 2.11-2.23 (m, 1H), 2.70 (s, 3H), 2.98 (dt, *J* = 14.0, 8.2 Hz, 1H), 3.11-3.19 (m, 1H), 4.67 (dd, *J* = 14.6, 4.7 Hz, 1H), 4.72 (dd, *J* = 14.6, 4.4 Hz, 1H), 6.05 (dd, *J* = 8.0, 3.9 Hz, 1H), 7.15 (s, 1H), 7.22-7.31 (m, 2H), 7.81-7.91 (m, 2H), 8.55 (s, 1H). ^{13}C NMR (125 MHz, $CDCl_3$): δ = 12.39, 33.69, 46.38, 55.61, 60.87, 79.93, 110.28, 116.35 (d, *J* = 21.7 Hz), 120.77, 127.57, 136.50, 145.81, 150.62, 162.26 (d, *J* = 243.2 Hz) 163.96. IR (nujol): v_{max} 1690. HRMS: calcd for $C_{18}H_{19}N_6O_3FNa^+$ 409.1395, found 409.1398.

5-methyl-1-((3*RS*,5*RS*)-2-methyl-3-((4-(4-pentylphenyl)-1*H*-1,2,3-triazol-1yl)methyl)isoxazolidin-5-yl)pyrimidine-2,4(1*H*,3*H*)-dione (14g): Yield 85%, white solid, m.p. 245-248 °C, ^{1}H NMR (300 MHz, $CDCl_3$): δ = 0.98 (t, *J* = 7.4 Hz, 3H), 1.29-1.37 (m, 2H), 1.61-1.66 (m, 4H), 1.73 (s, 3H), 2.16-2.34 (m, 1H), 2.63 (t, *J* = 7.4, 2H), 2.76 (s, 3H), 3.09 (dt, *J* = 14.3, 7.9 Hz, 1H), 3.12-3.31 (m, 1H), 4.52 (dd, *J* = 14.5, 4.6 Hz, 1H), 4.67 (dd, *J* = 14.5, 5.0 Hz, 1H), 6.14 (dd, *J* = 7.7, 4.0 Hz, 1H), 7.23 (d, *J* = 8.0 Hz, 2H), 7.26 (s, 1H), 7.77 (d, *J* = 8.0 Hz, 2H), 7.72 (s, 1H), 8.18 (bs, 1H). ^{13}C NMR (126 MHz, DMSO): δ = 7.90, 12.16, 22.60, 30.75, 31,09, 32.45, 34.40, 47.15, 54.43, 62.87, 79.80, 109.65, 120.55, 121.36, 124.99, 127.95, 138.35, 147.67, 150.05, 169.29. IR (nujol): v_{max} 1698. HRMS: calcd for $C_{23}H_{30}N_6O_3Na^+$ 461.2272, found 461.2277.

^{1}H NMR (500 MHz, CDCl3) of compound **9**

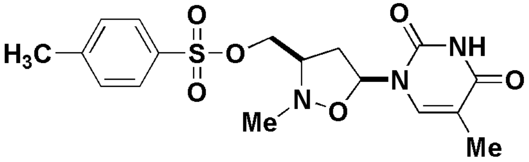

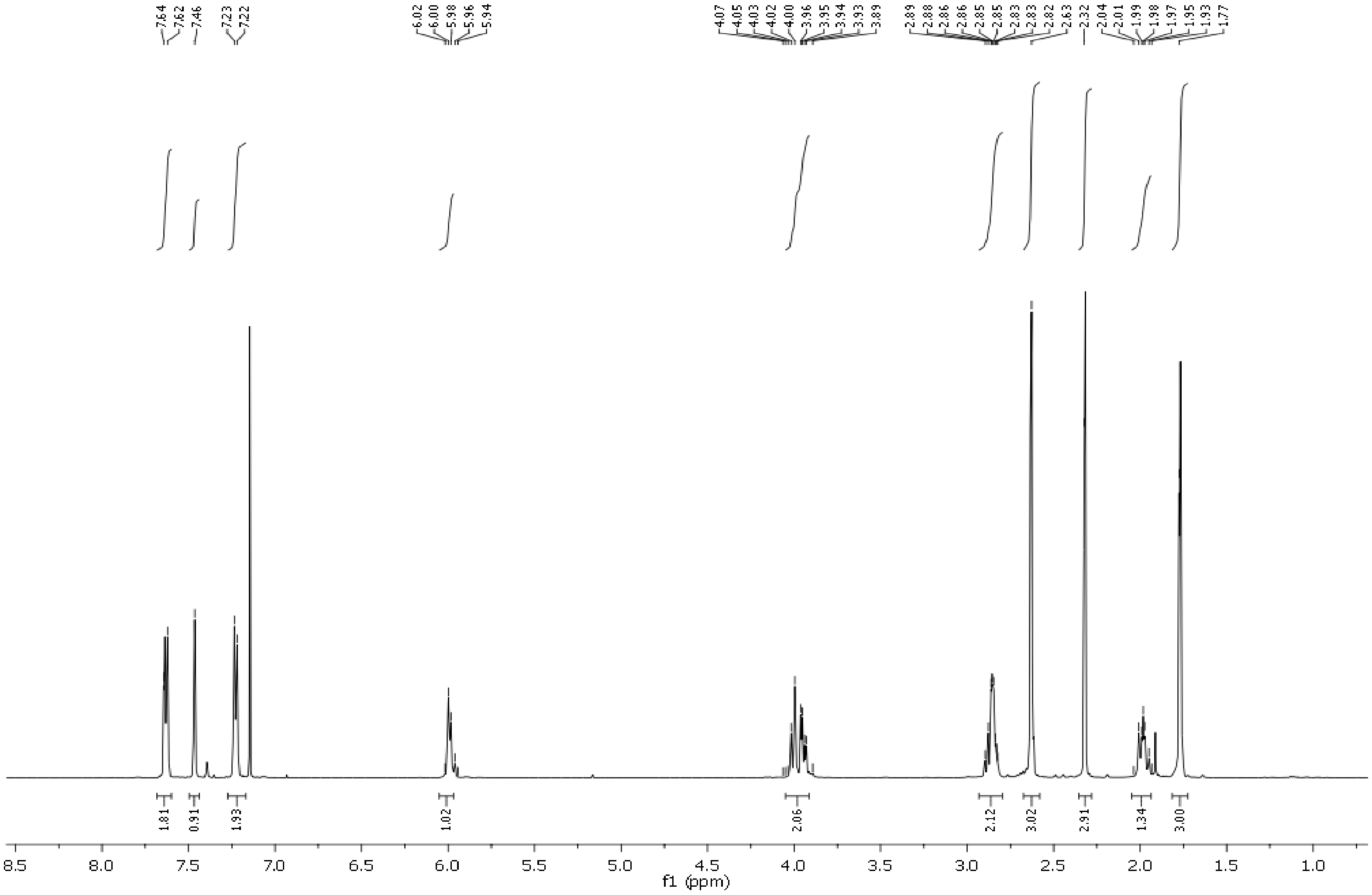

^{13}C NMR (126 MHz, CDCl3) of compound **9**

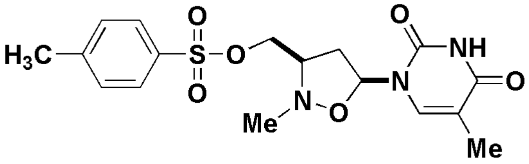

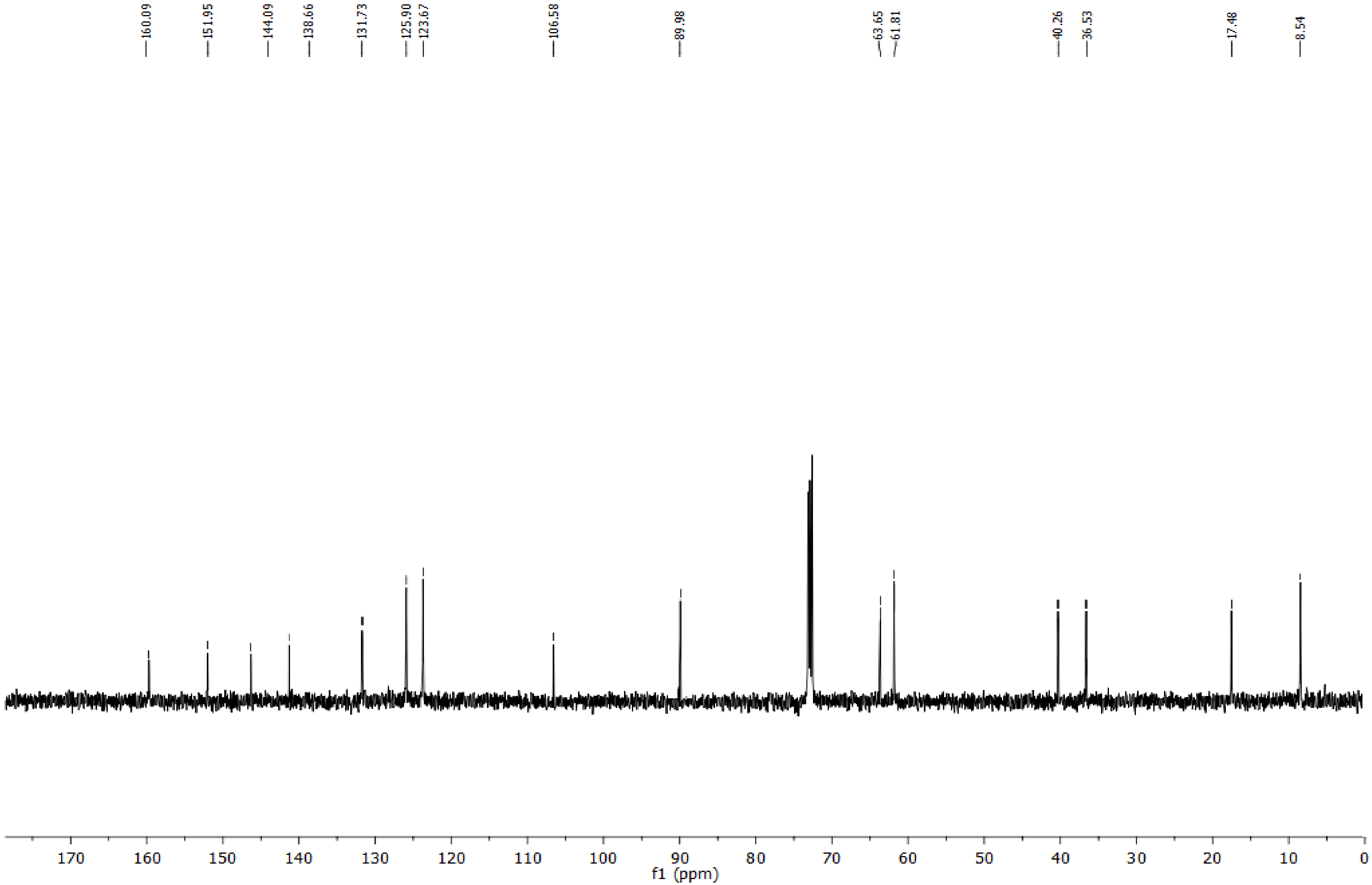

^{1}H NMR (300 MHz, CDCl3) of compound **10**

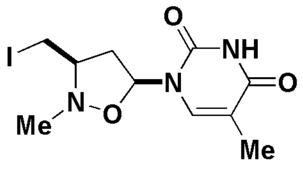

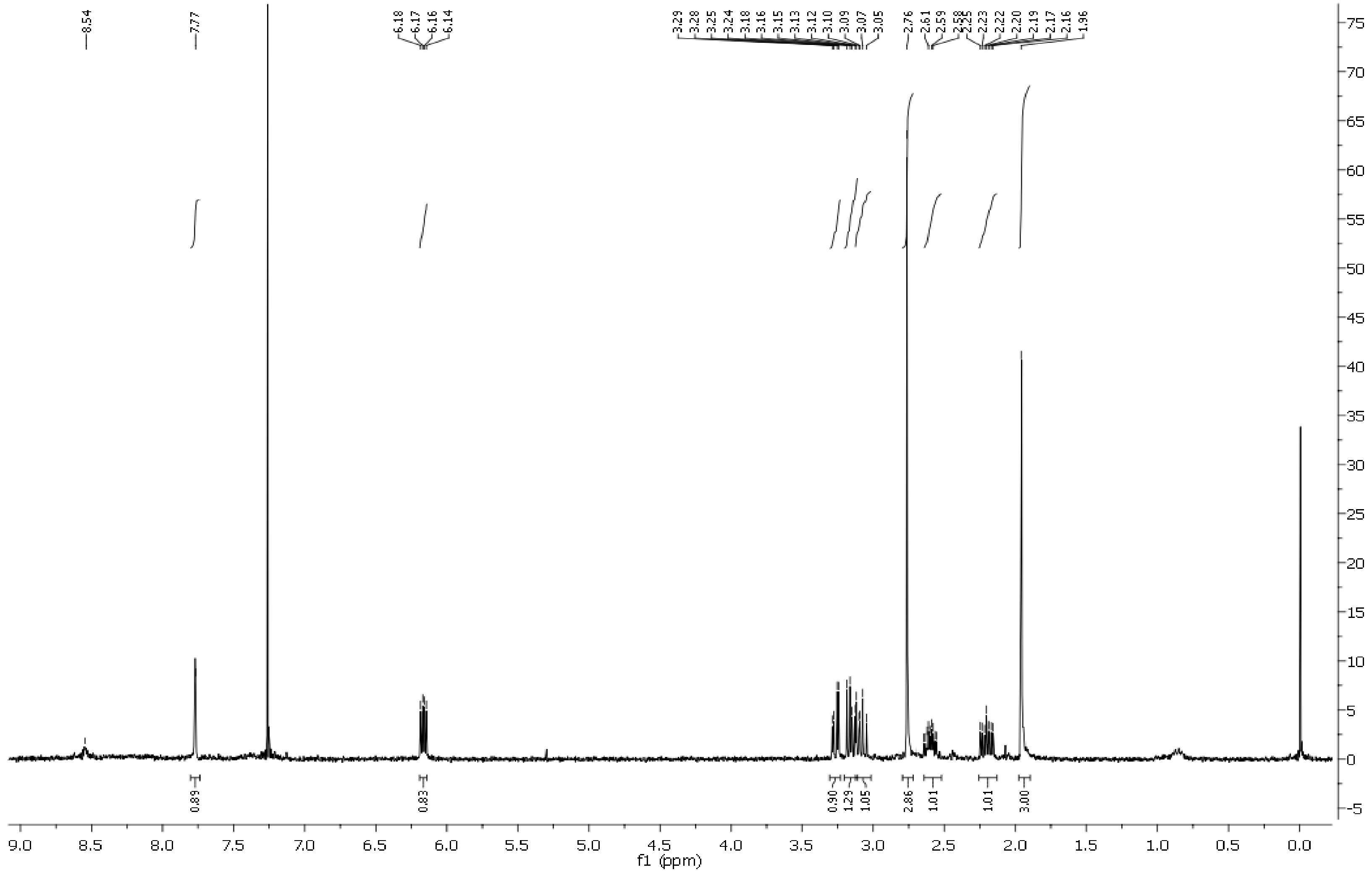

^{13}C NMR (126 MHz, CDCl3) of compound **10**

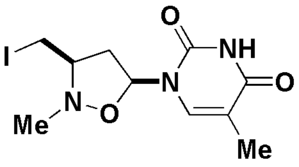

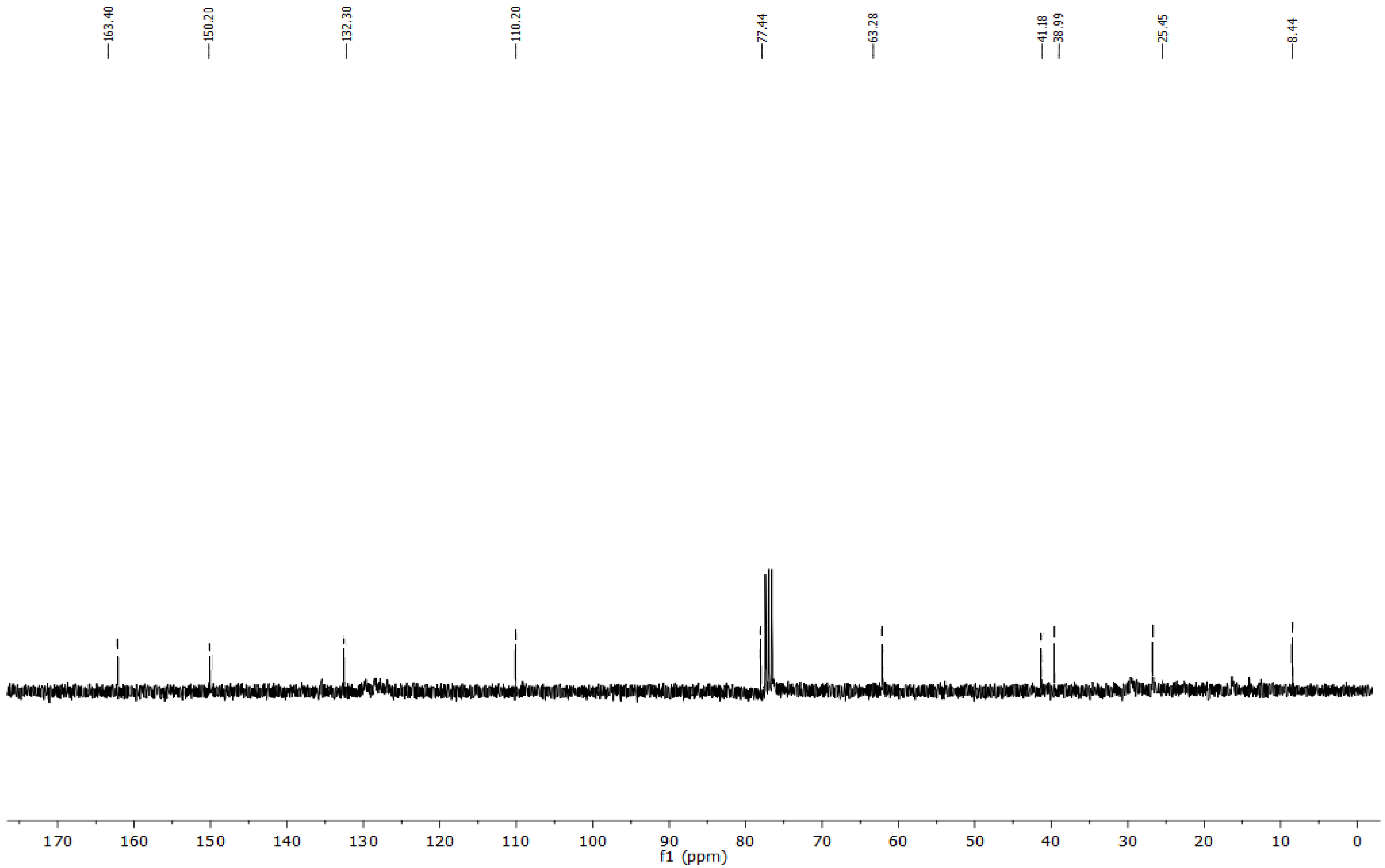

^{1}H NMR (300 MHz, CDCl3) of compound **11**

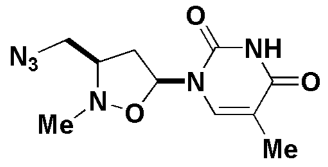

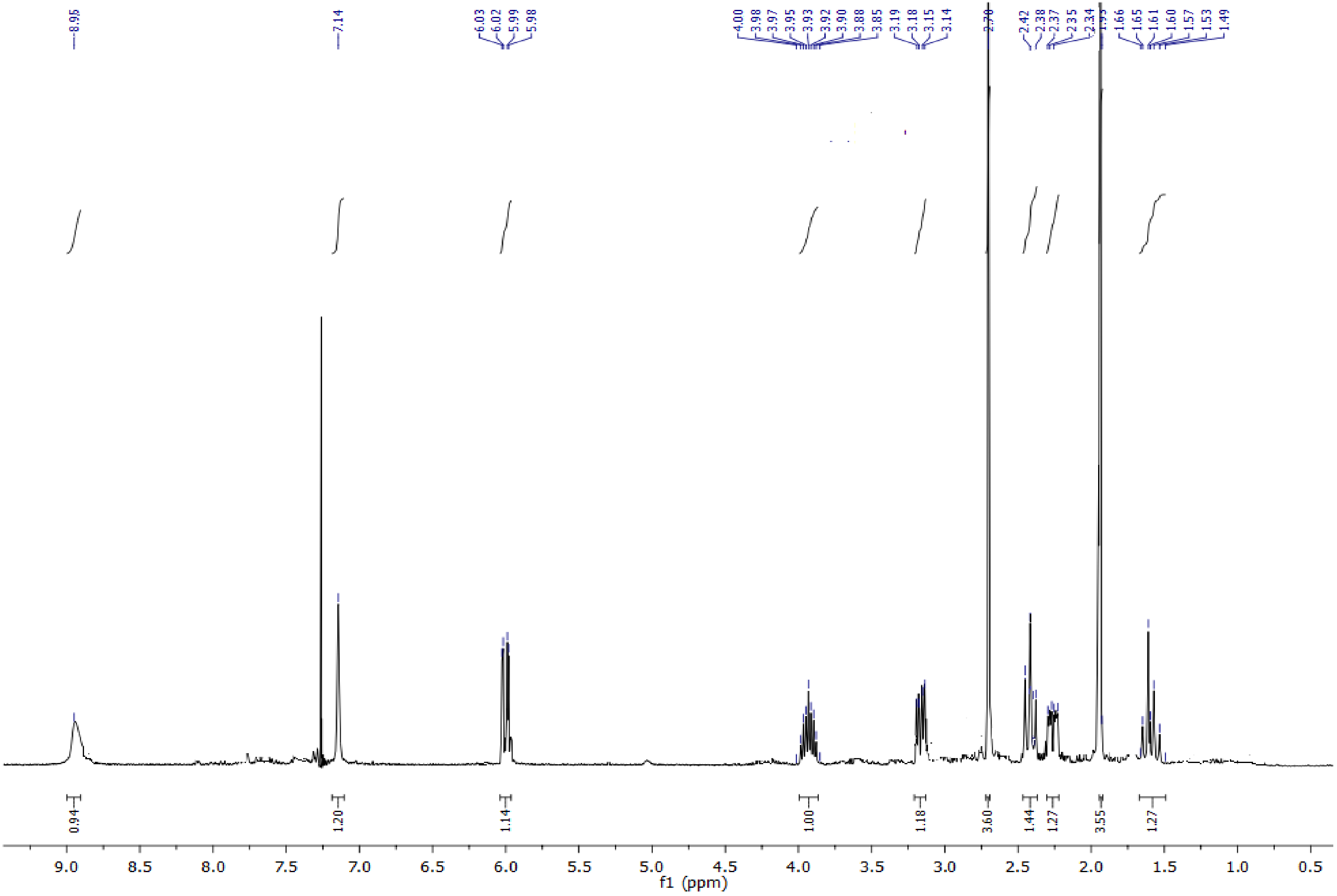

^{13}C NMR (126 MHz, CDCl3) of compound **11**

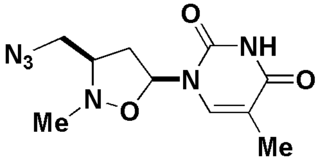

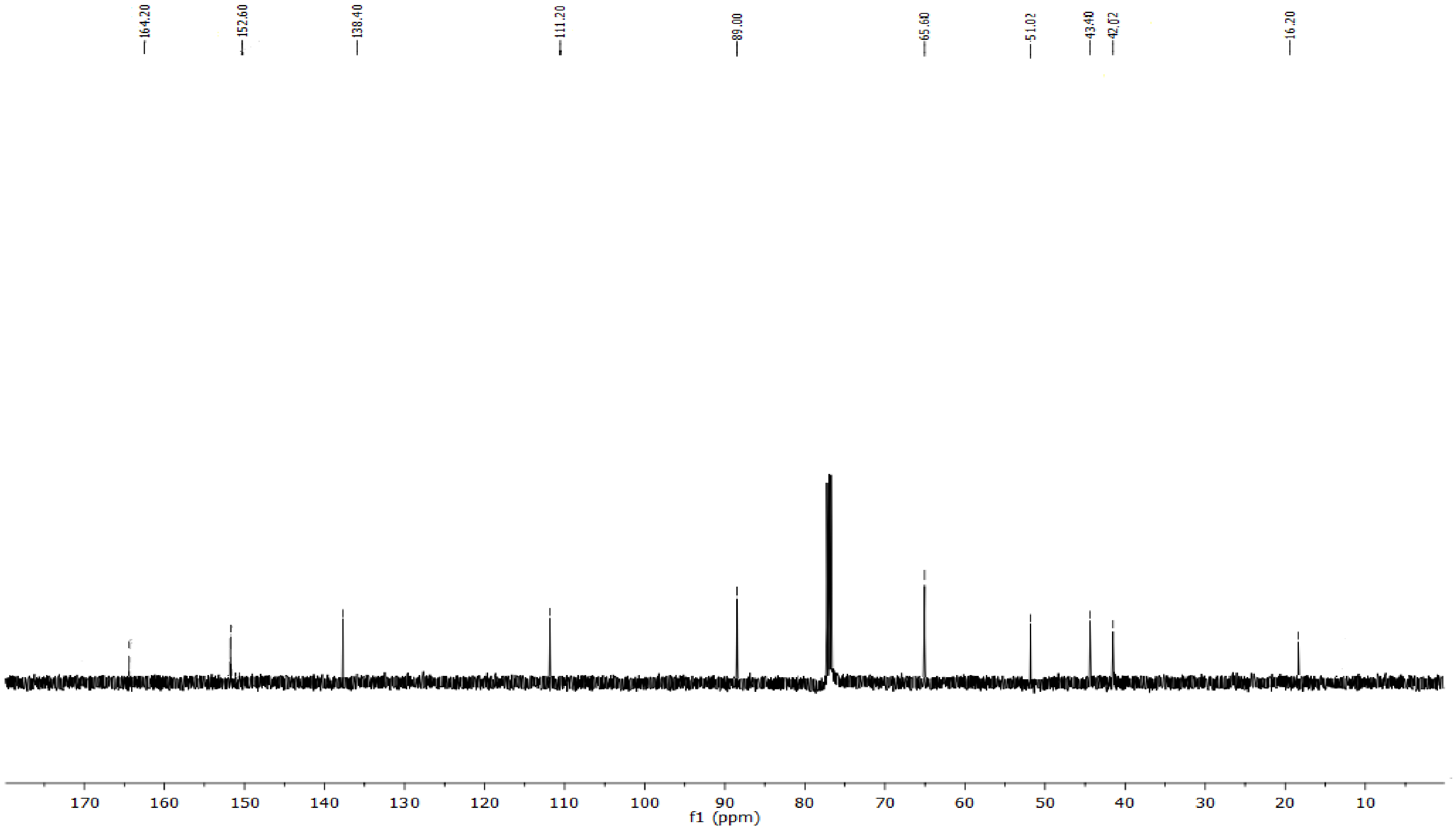

^{1}H NMR (500 MHz, CDCl3) of compound **12**

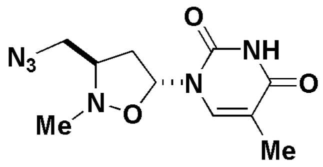

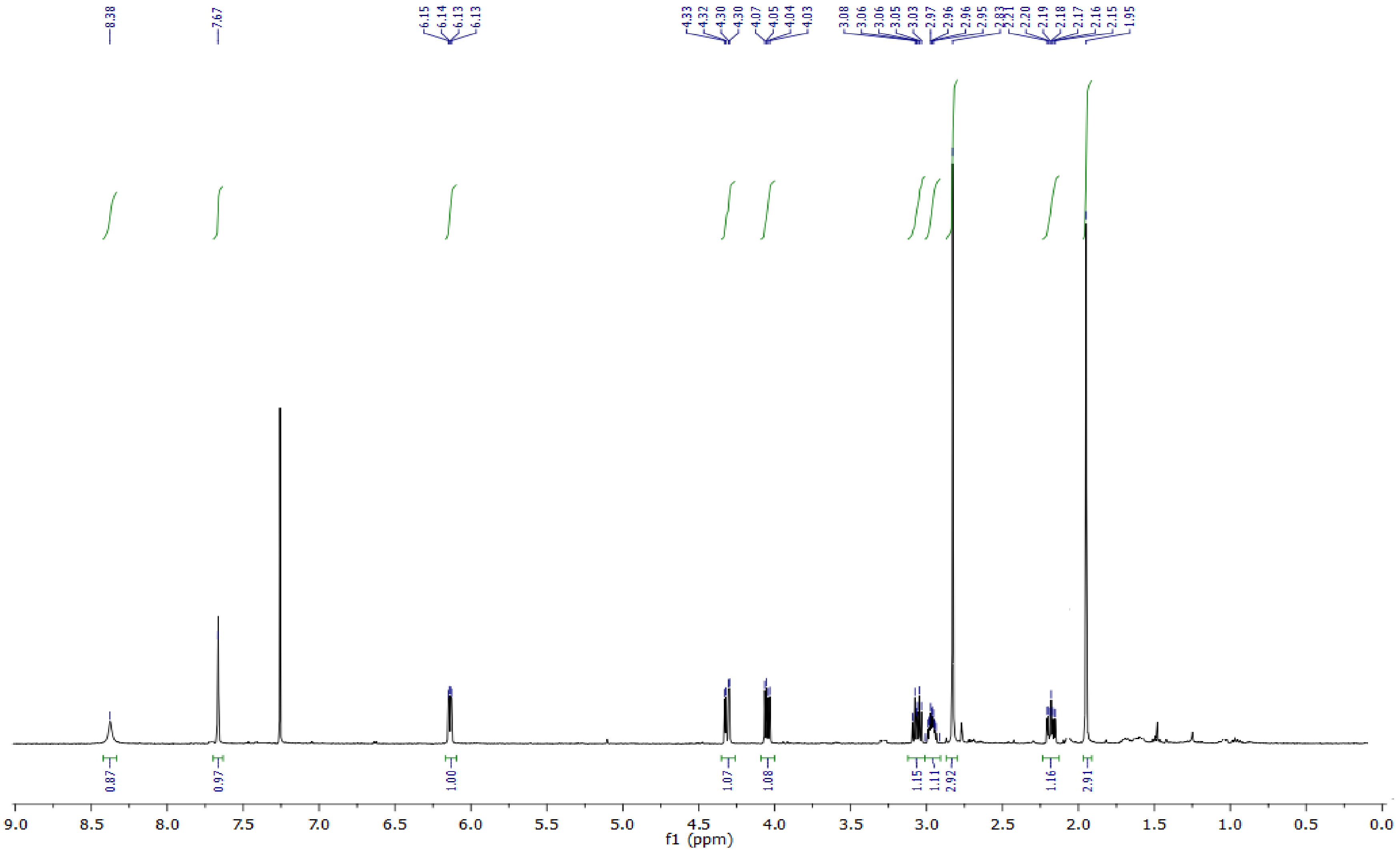

^{13}C NMR (126 MHz, CDCl3) of compound **12**

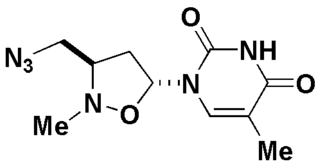

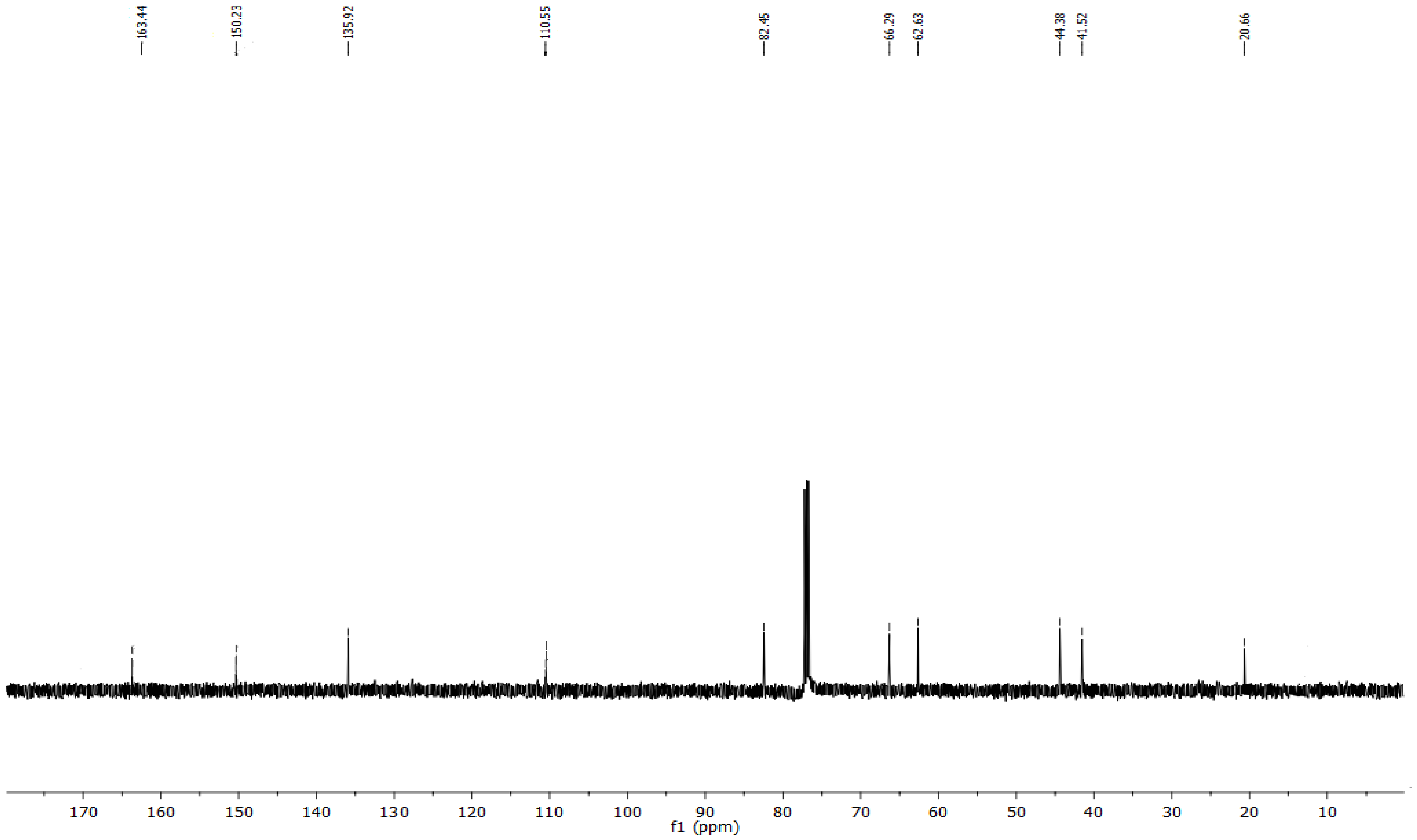

[1] H NMR (500 MHz, DMSO) of compound **13a**

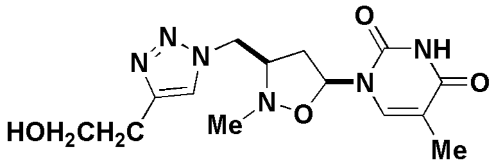

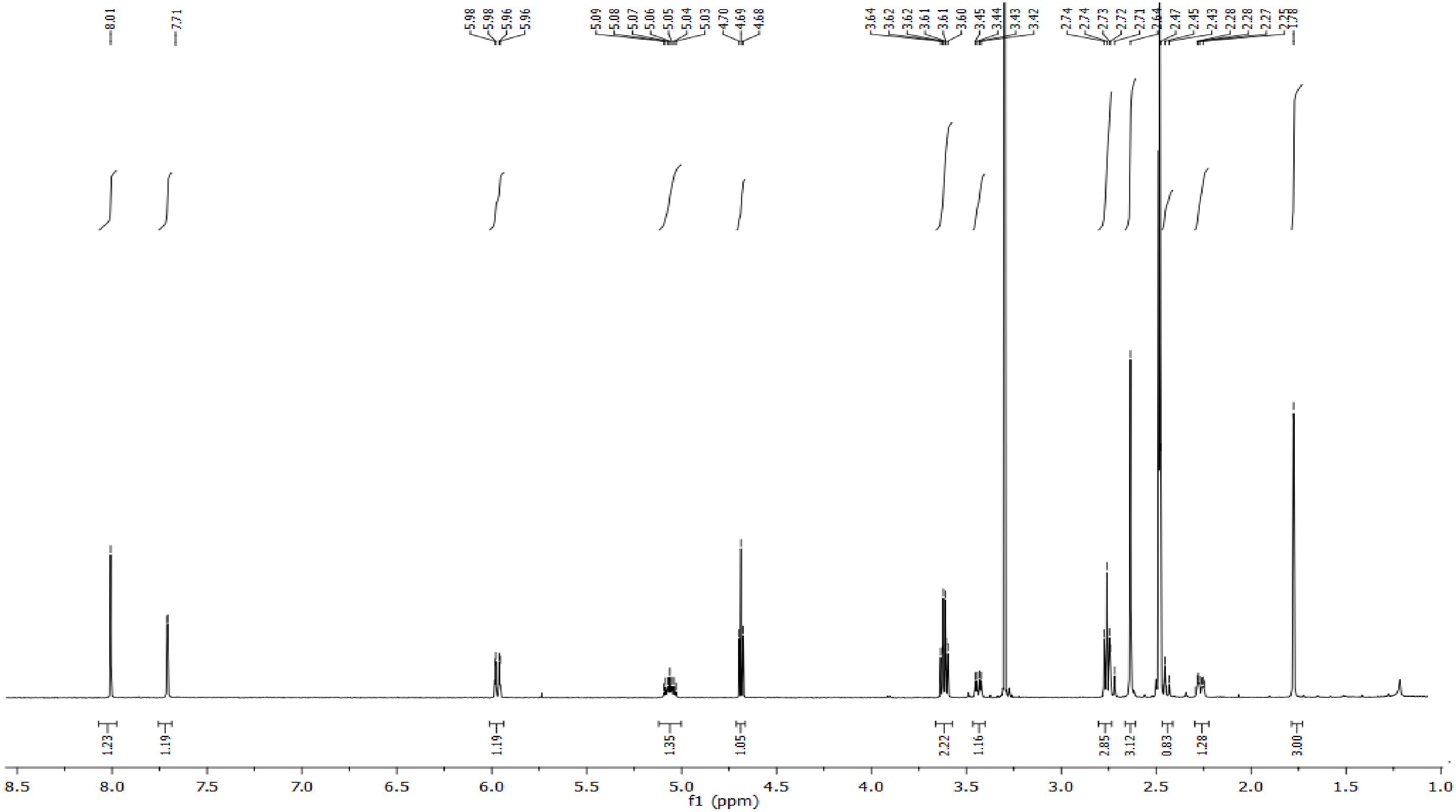

^{13}C NMR (126 MHz, DMSO) of compound **13a**

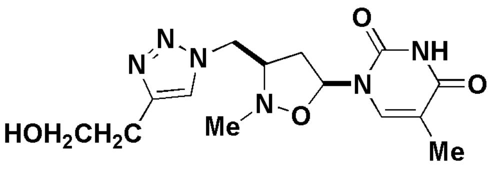

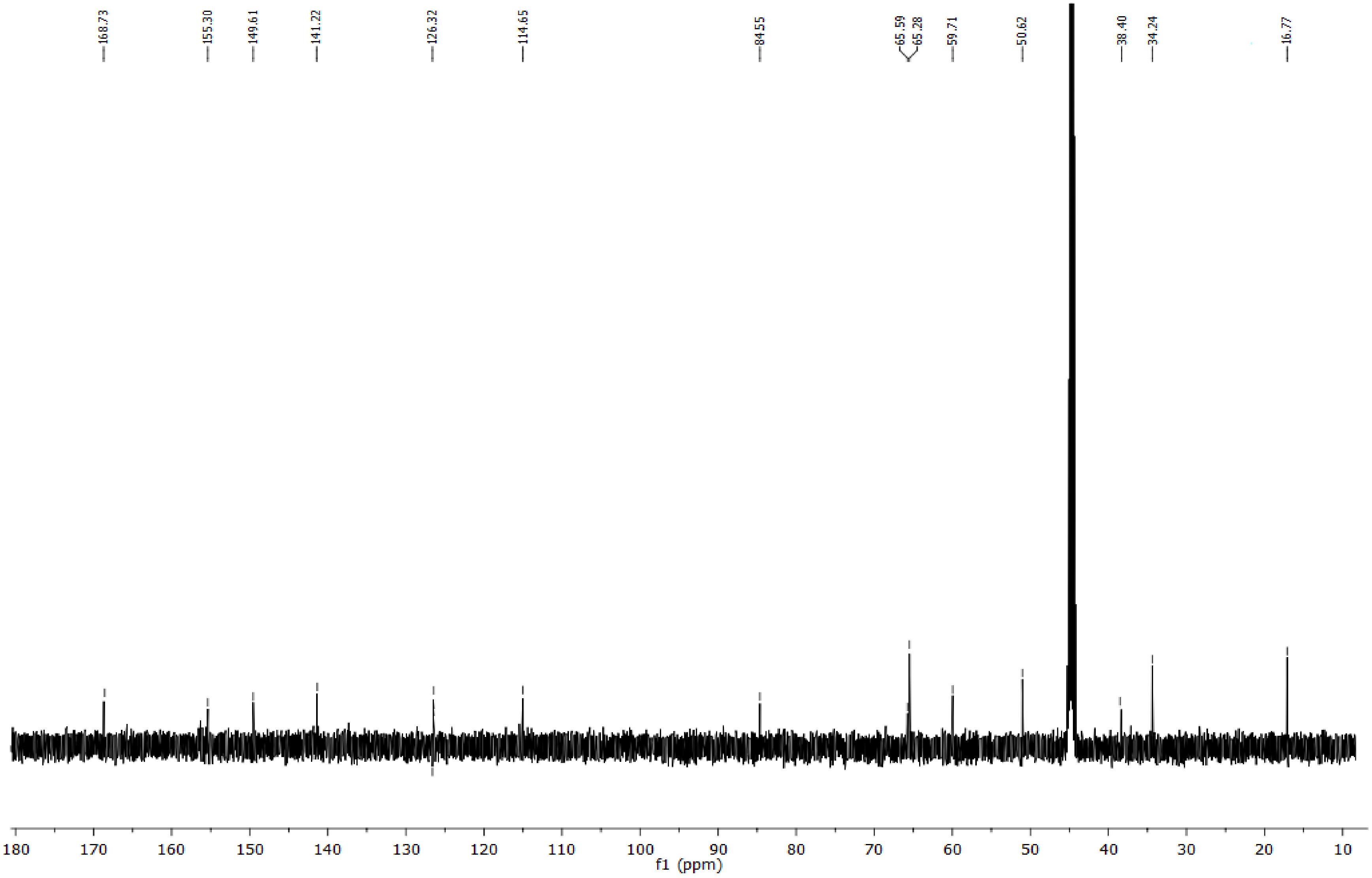

[1] H NMR (500 MHz, DMSO) of compound **13b**

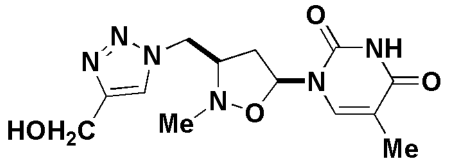

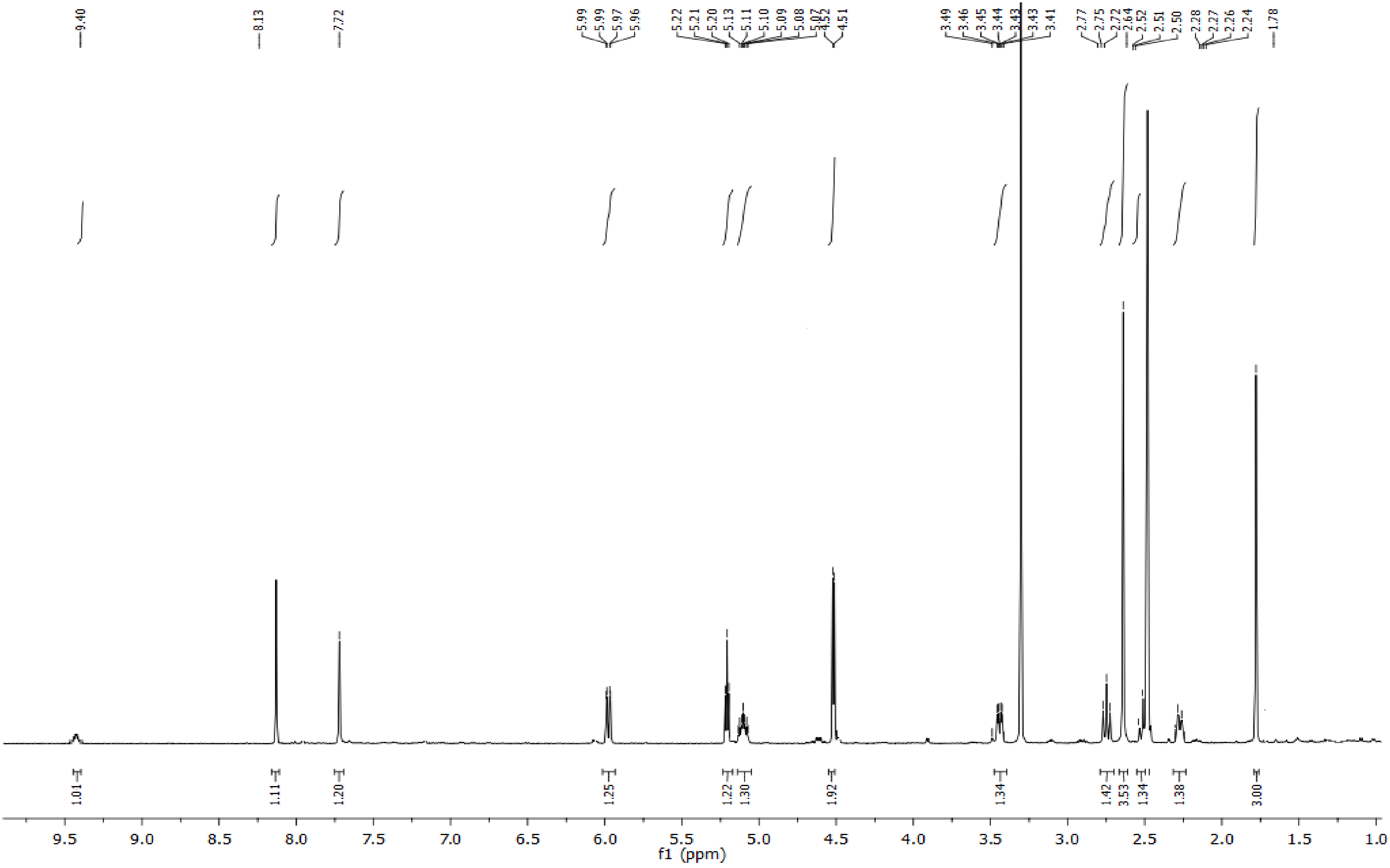

^{13}C NMR (126 MHz, DMSO) of compound **13b**

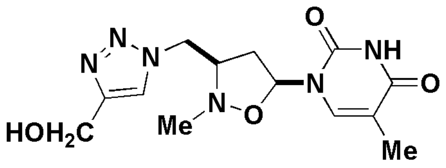

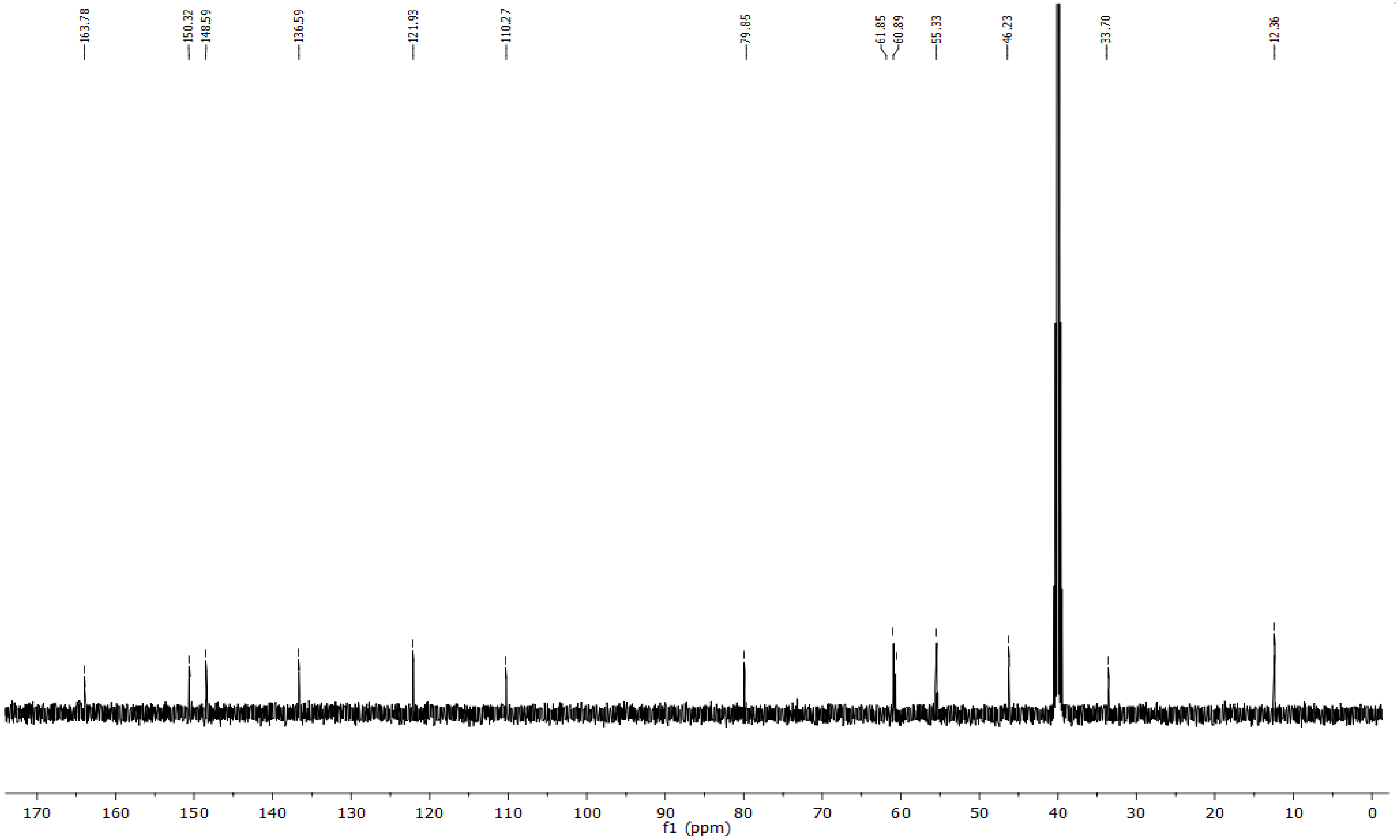

H NMR (500 MHz, CDCl3) of compound **13c**

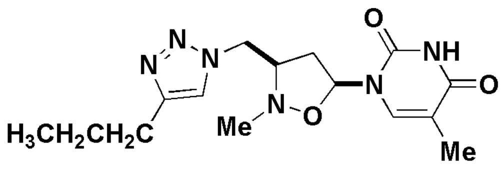

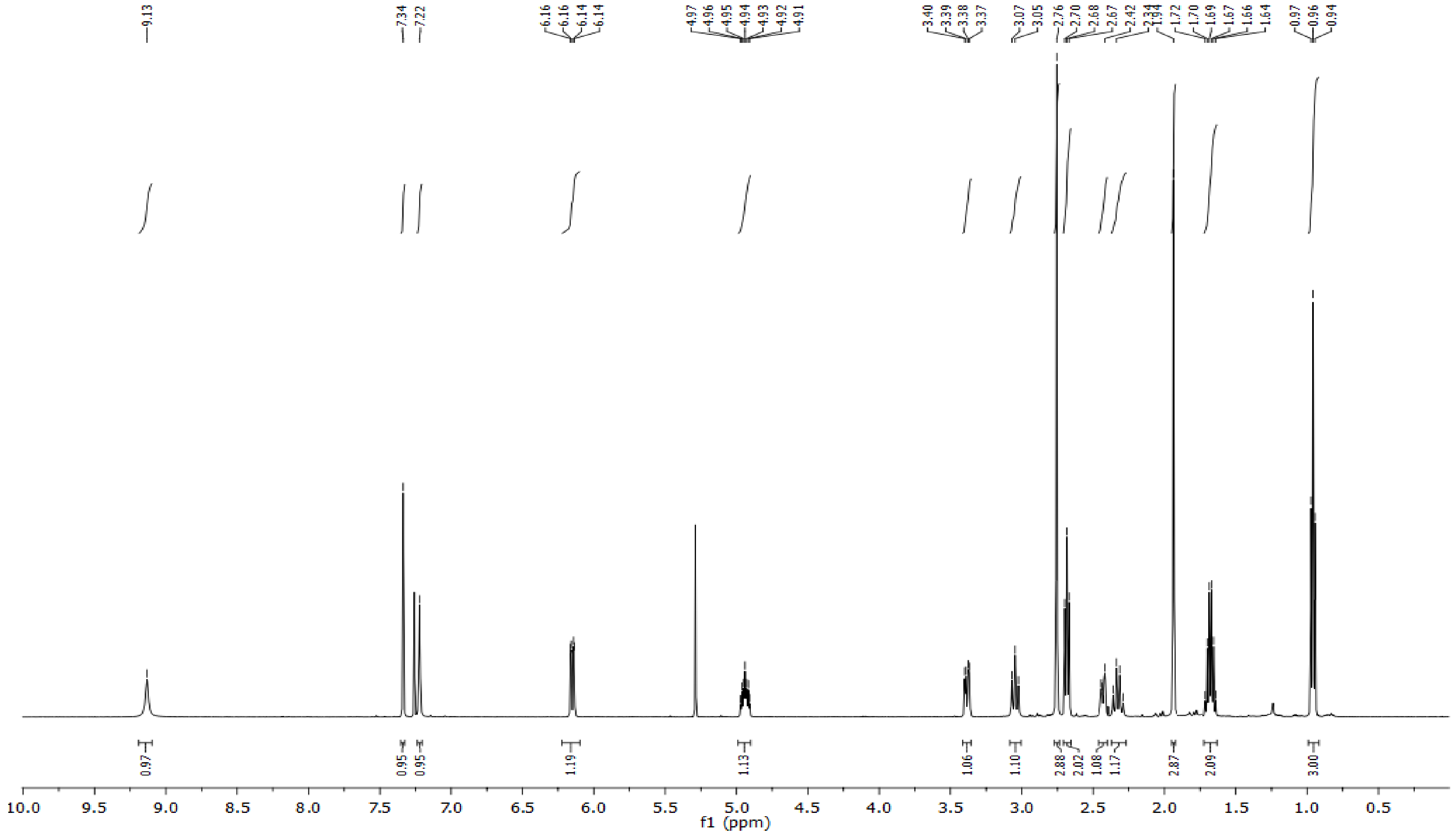

[13] C NMR (126 MHz, CDCl3) of compound **13c**

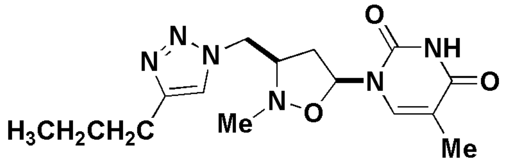

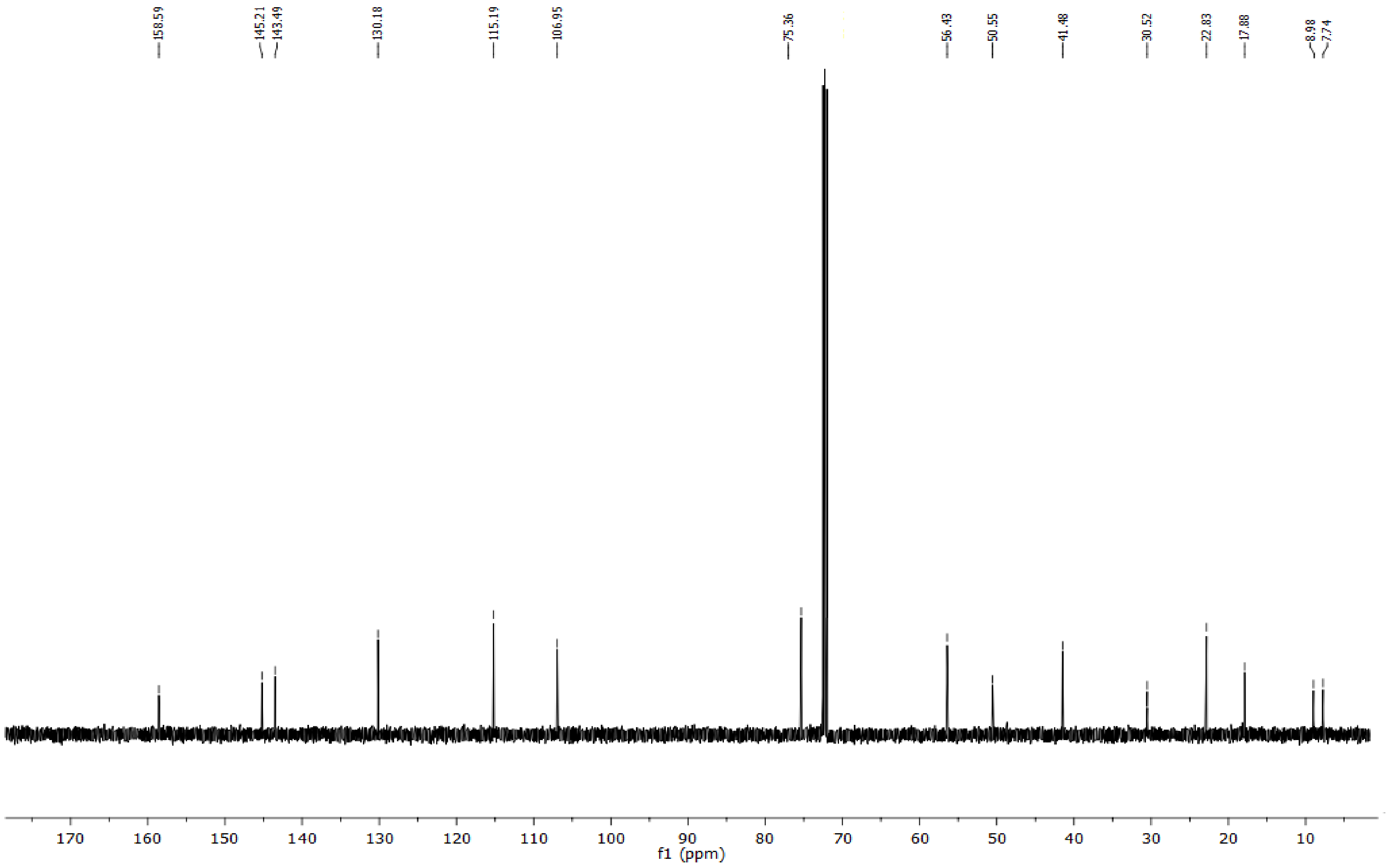

[1] H NMR (300 MHz, CDCl3) of compound **13d**

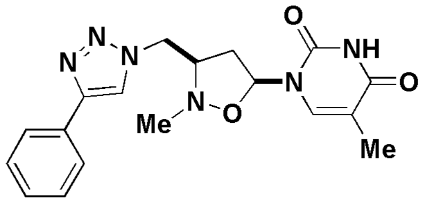

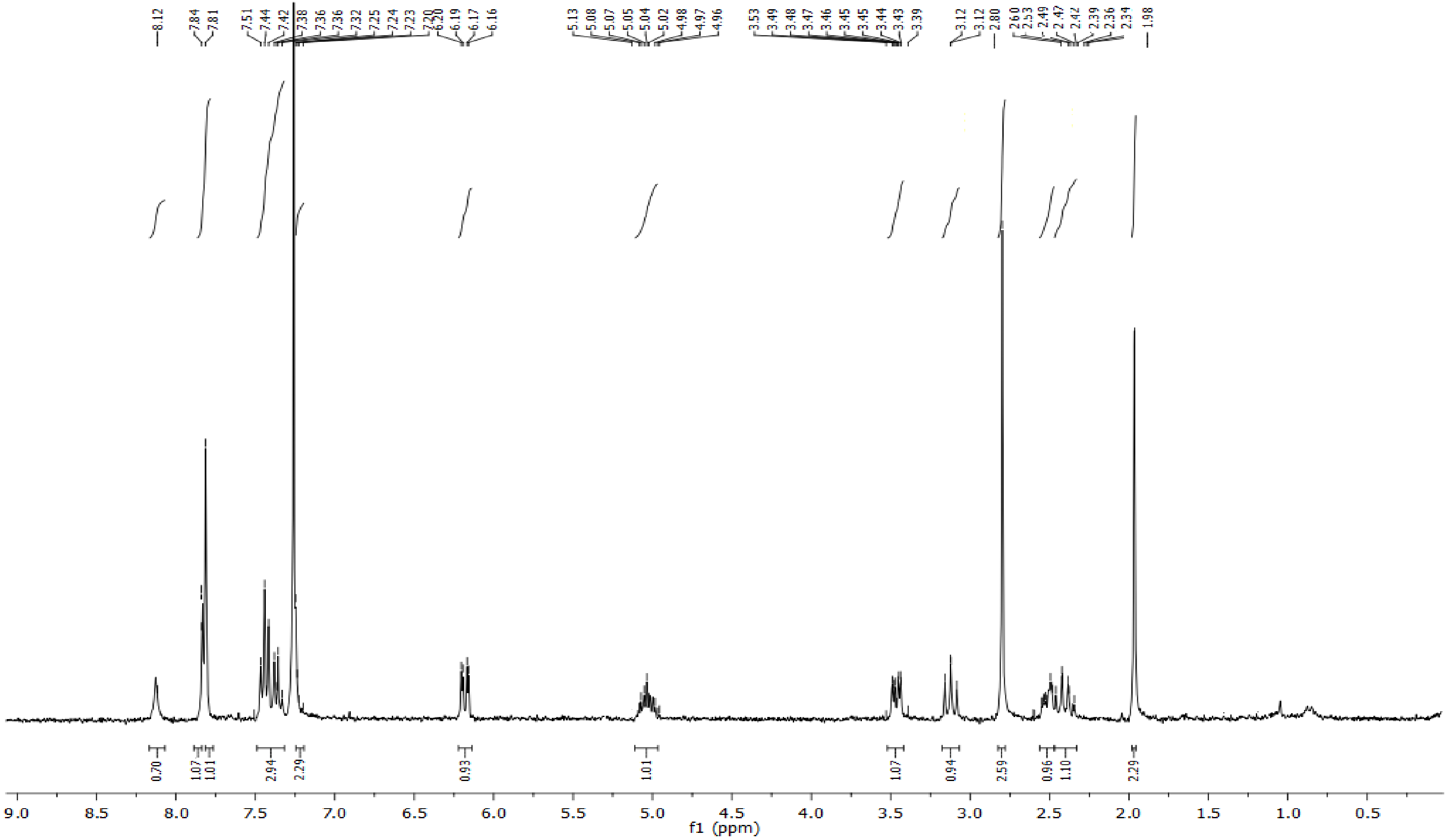

^{13}C NMR (126 MHz, CDCl3) of compound **13d**

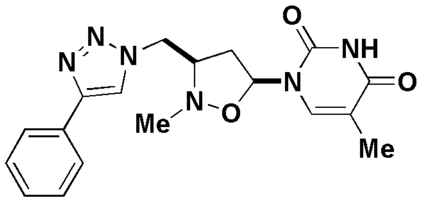

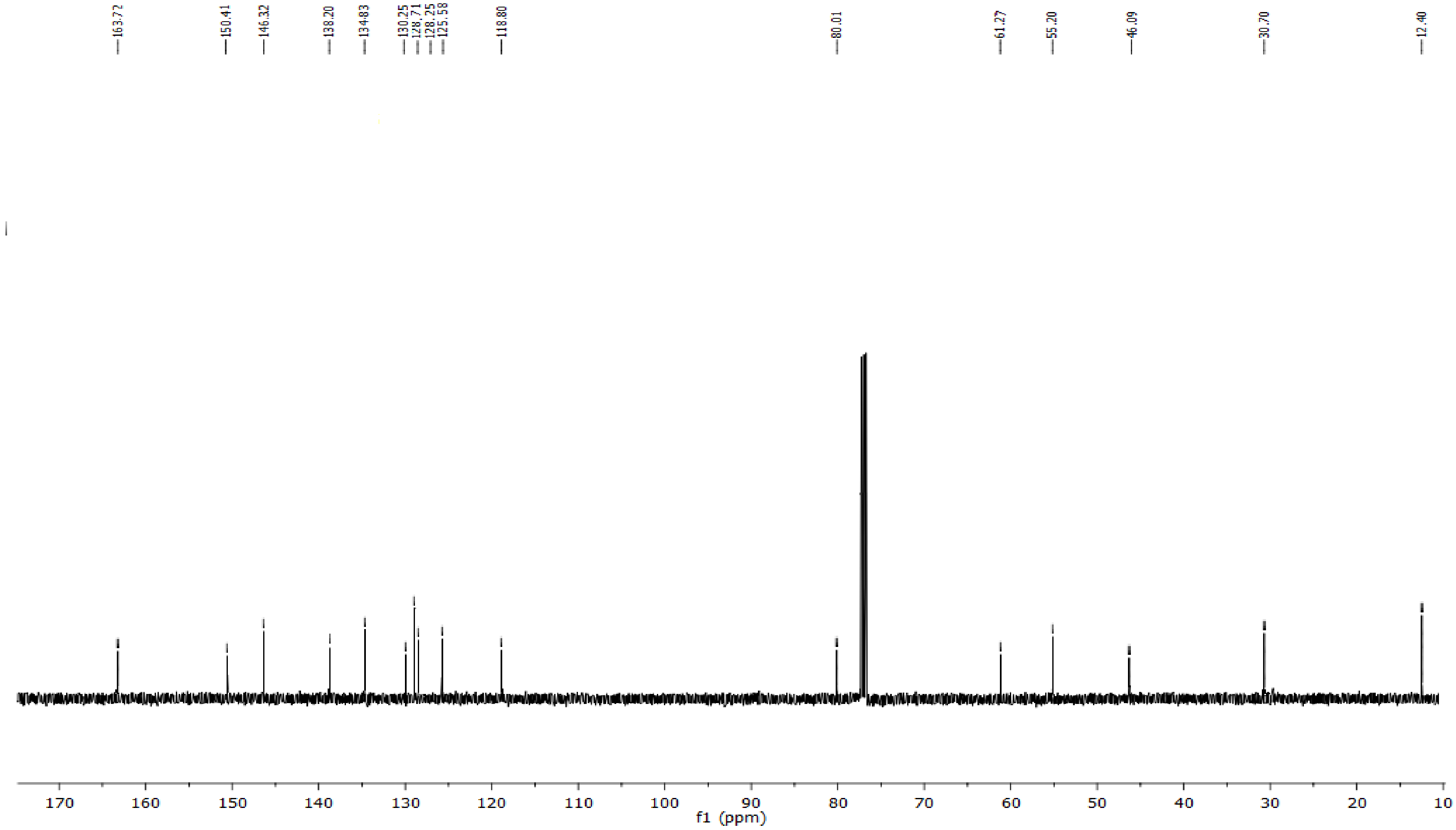

[1] H NMR (500 MHz, DMSO) of compound **13e**

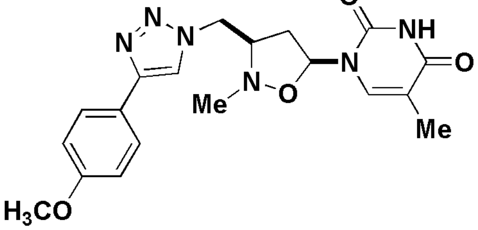

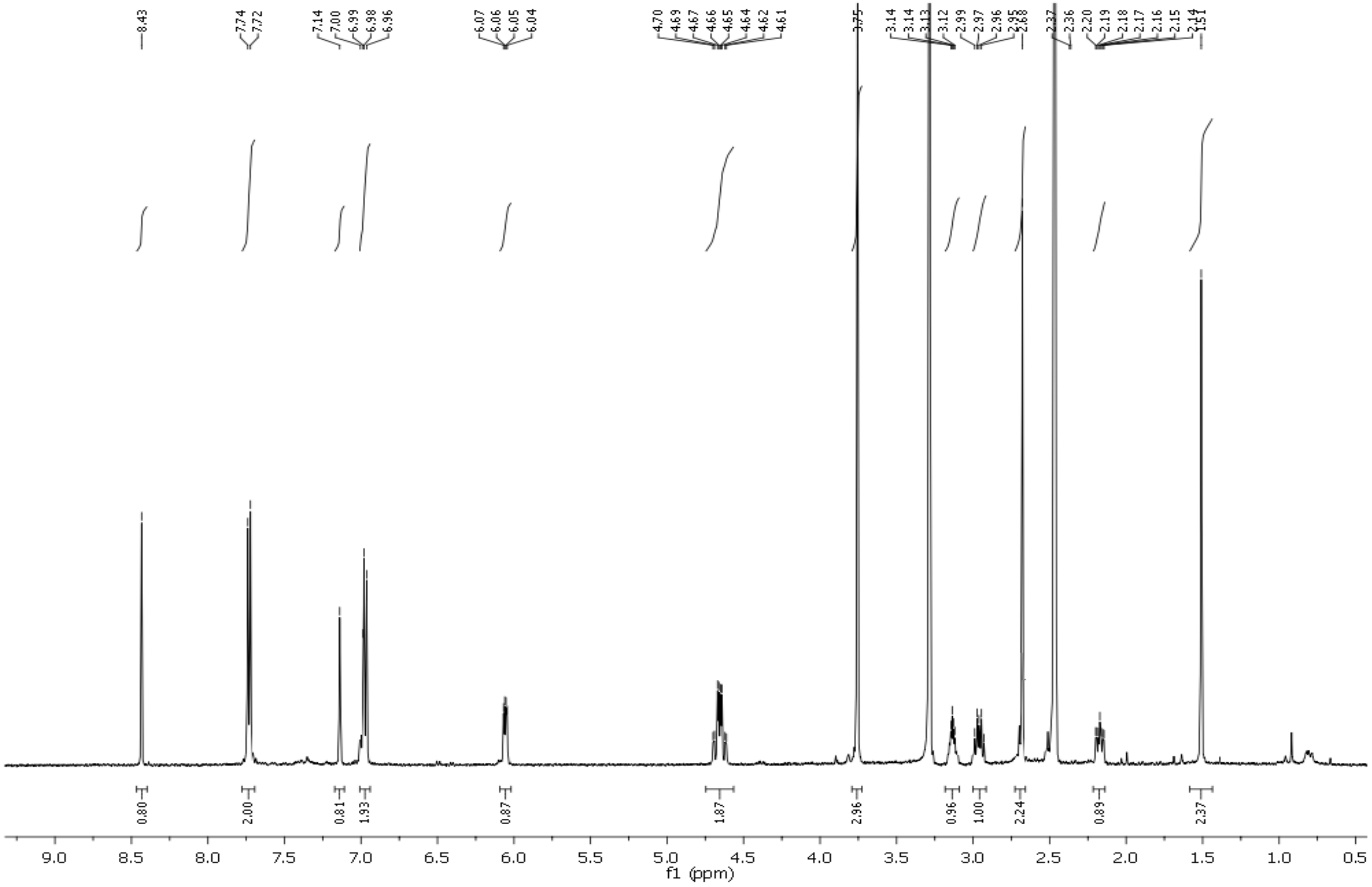

^{13}C NMR (126 MHz, DMSO) of compound **13e**

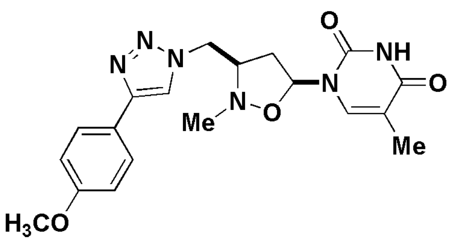

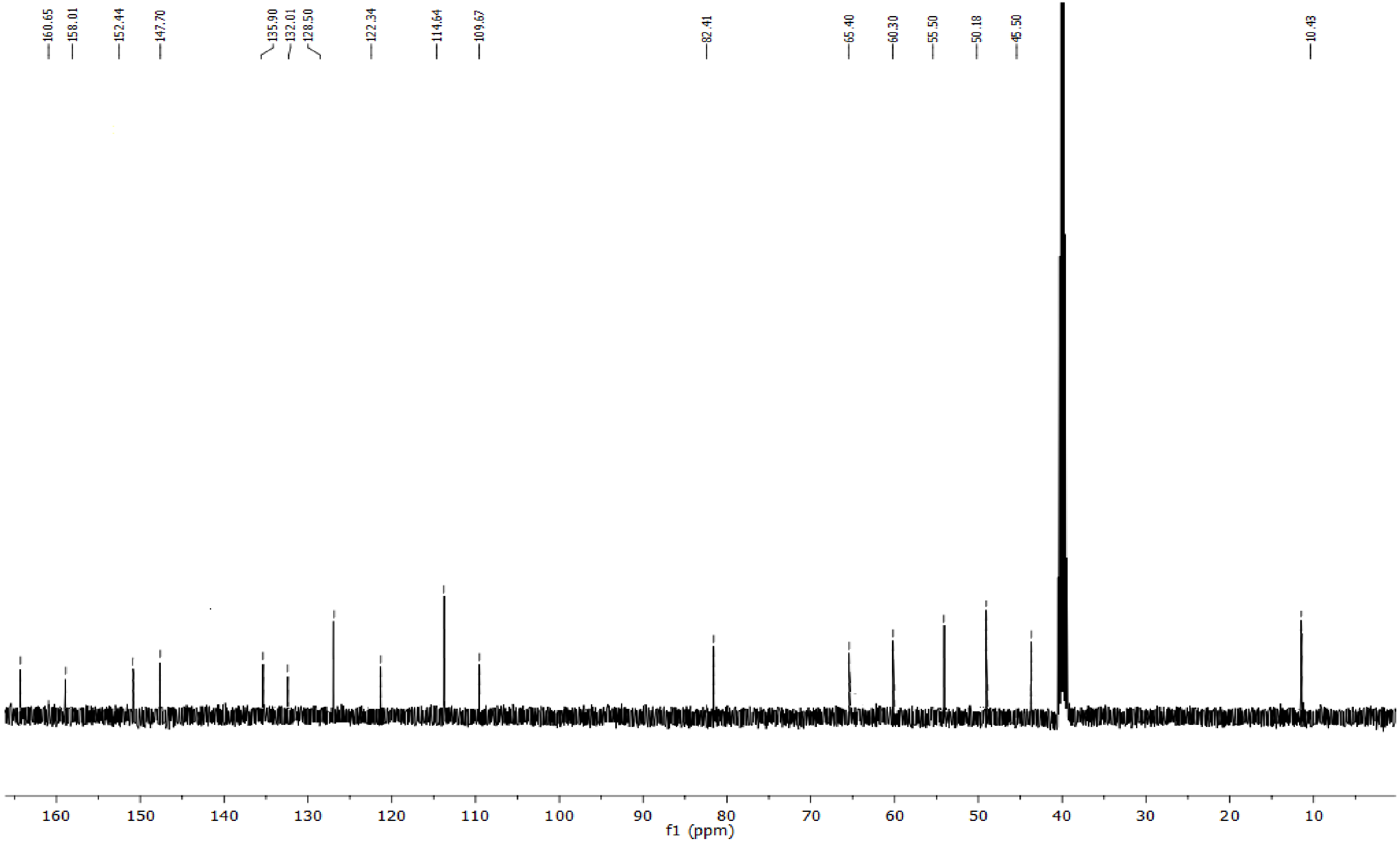

[1] H NMR (500 MHz, CDCl3) of compound **13f**

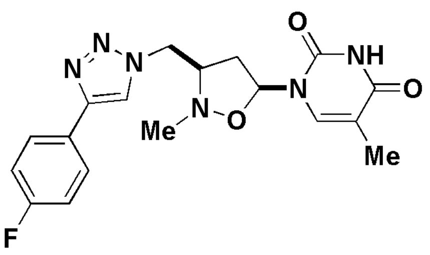

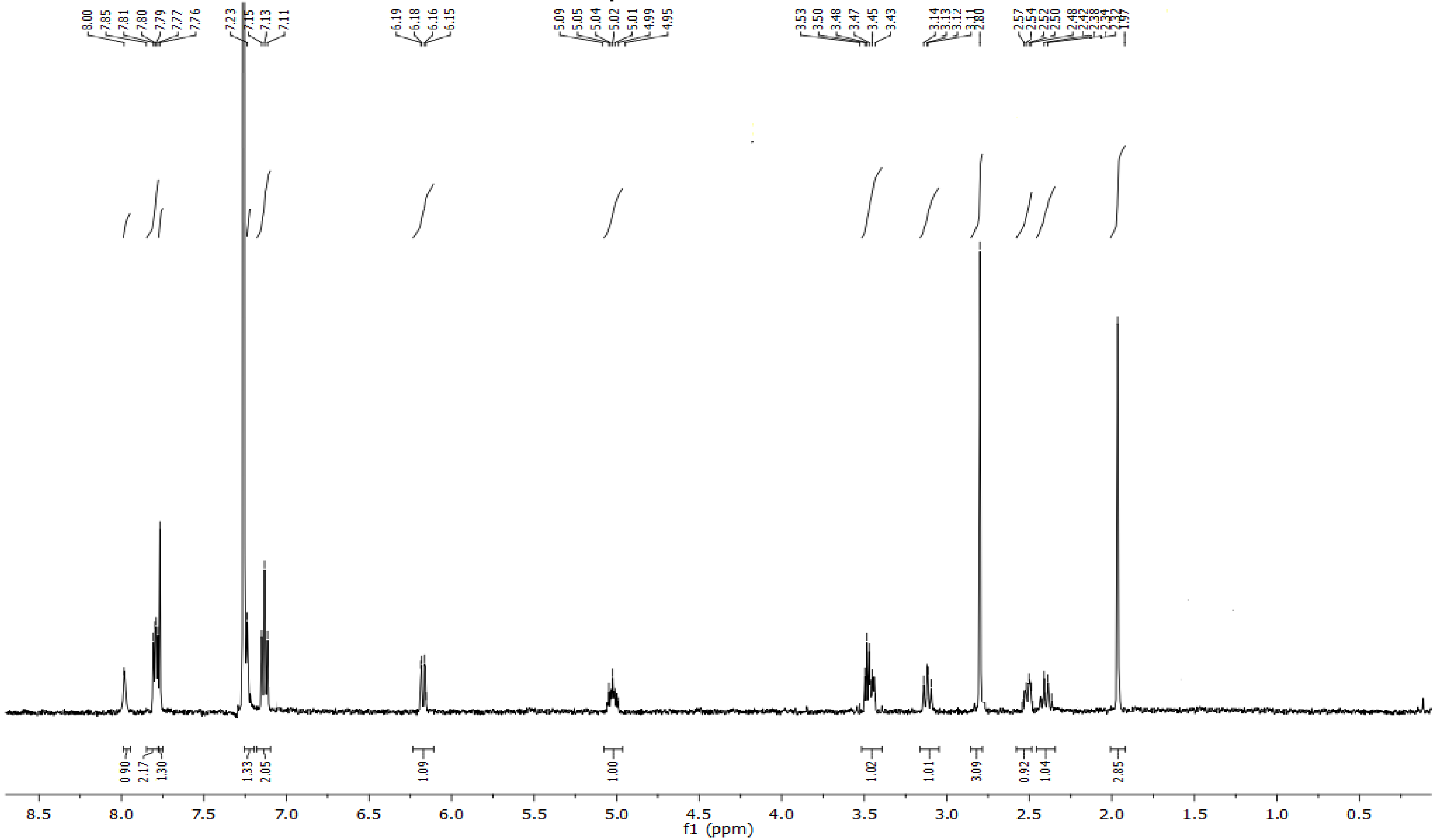

^{13}C NMR (126 MHz, DMSO) of compound **13f**

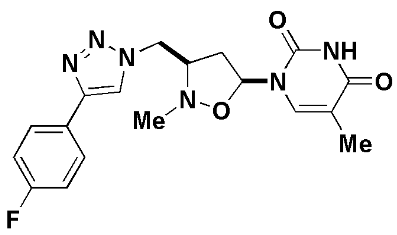

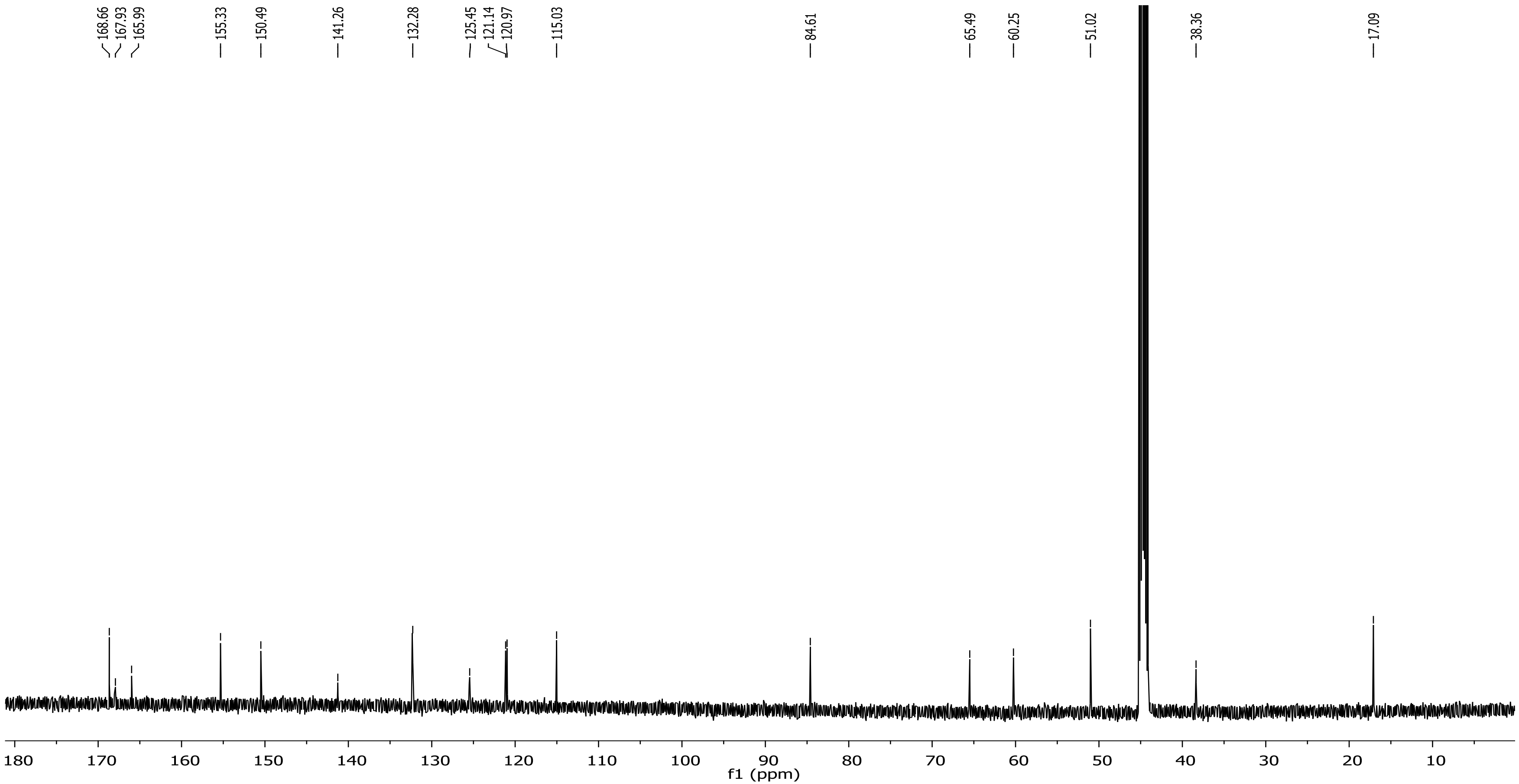

[1] H NMR (300 MHz, CDCl3) of compound **13g**

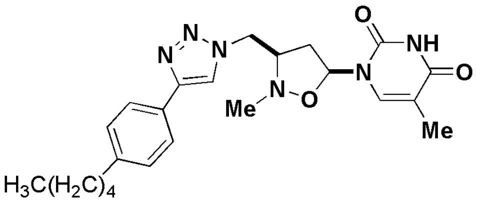

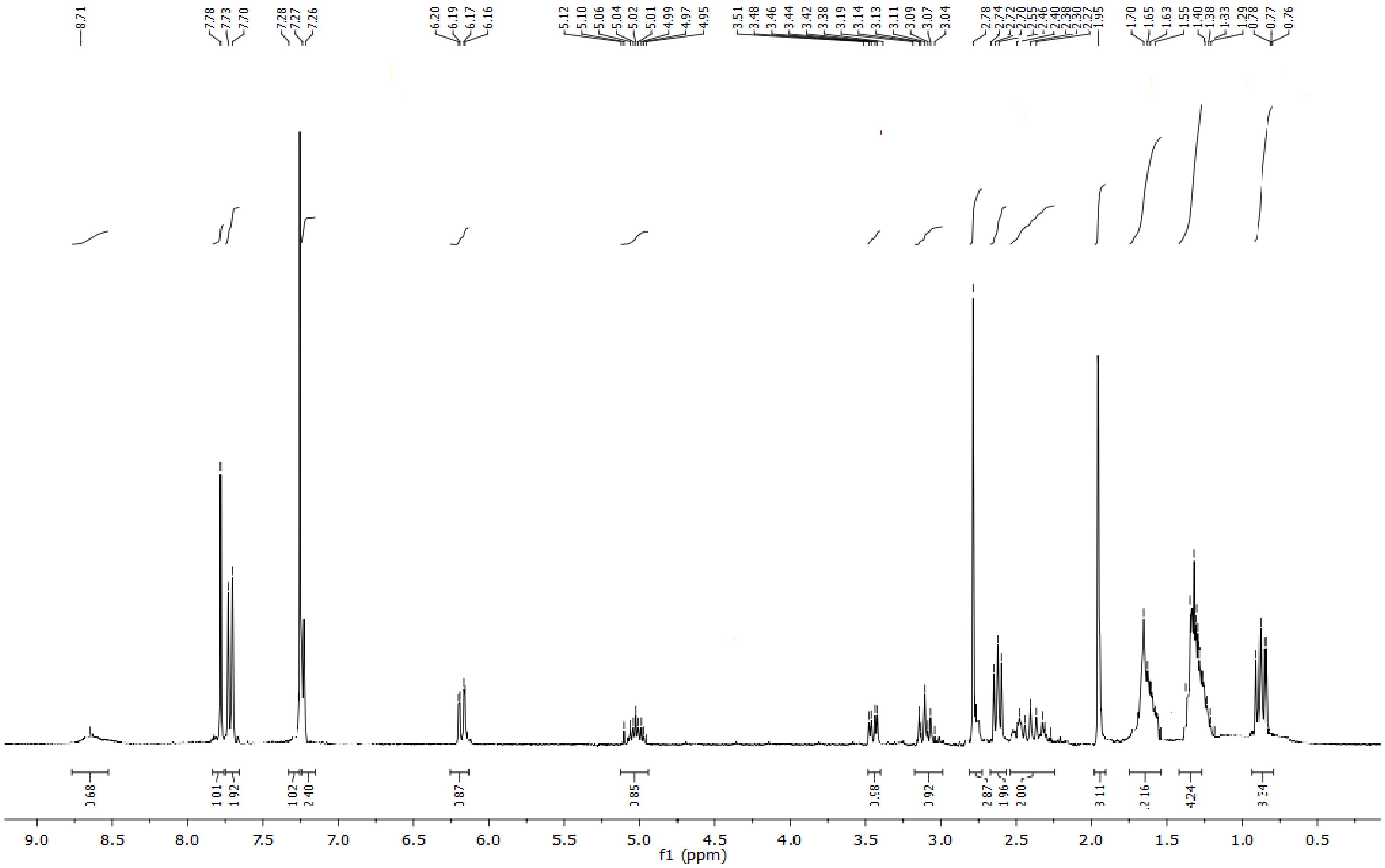

[13] C NMR (126 MHz, DMSO) of compound **13g**

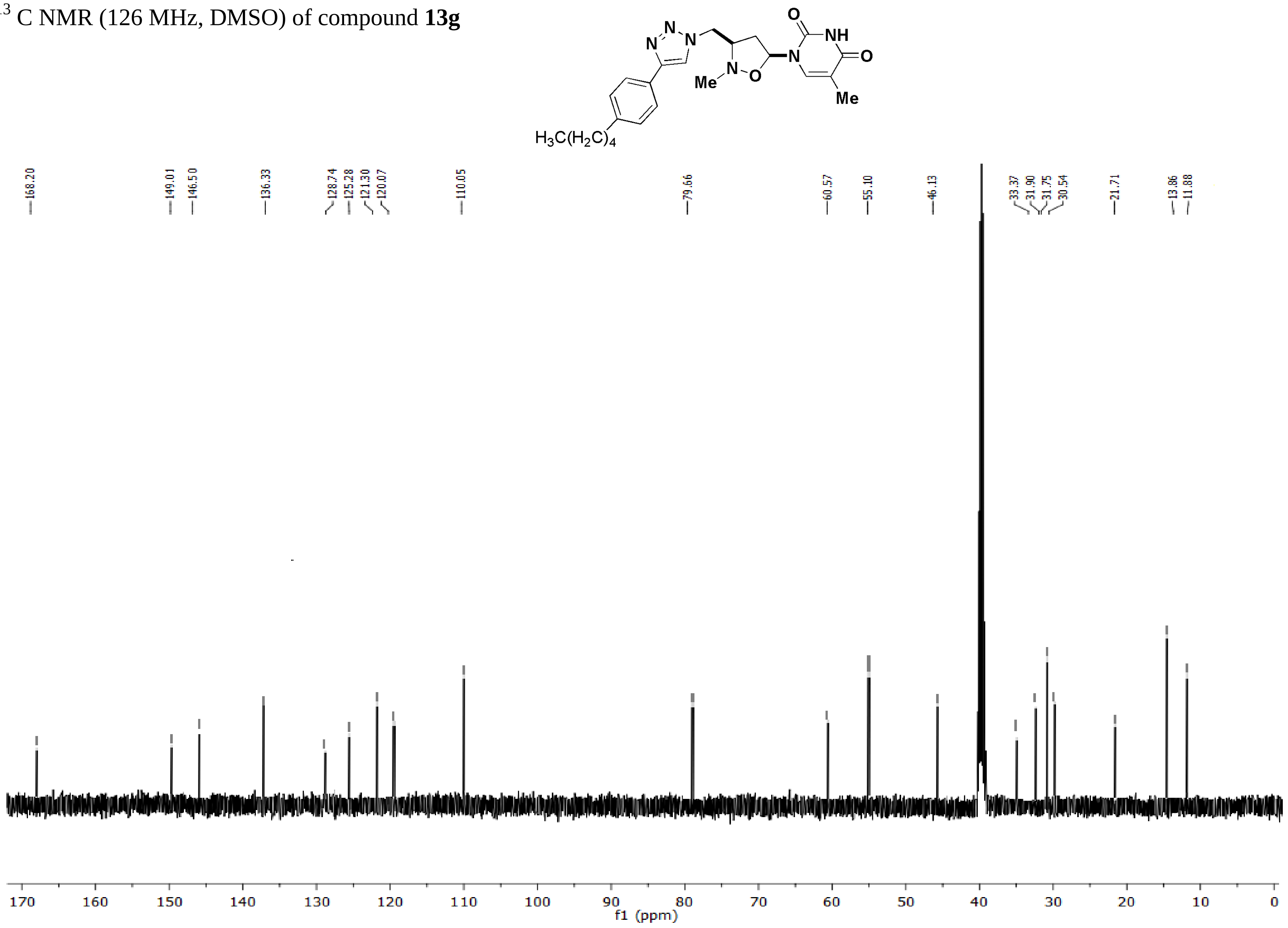

[1] H NMR (500 MHz, CDCl3) of compound **14a**

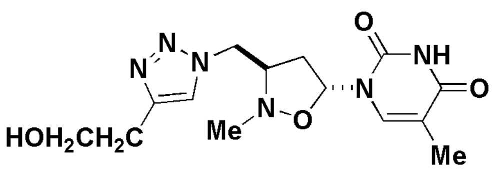

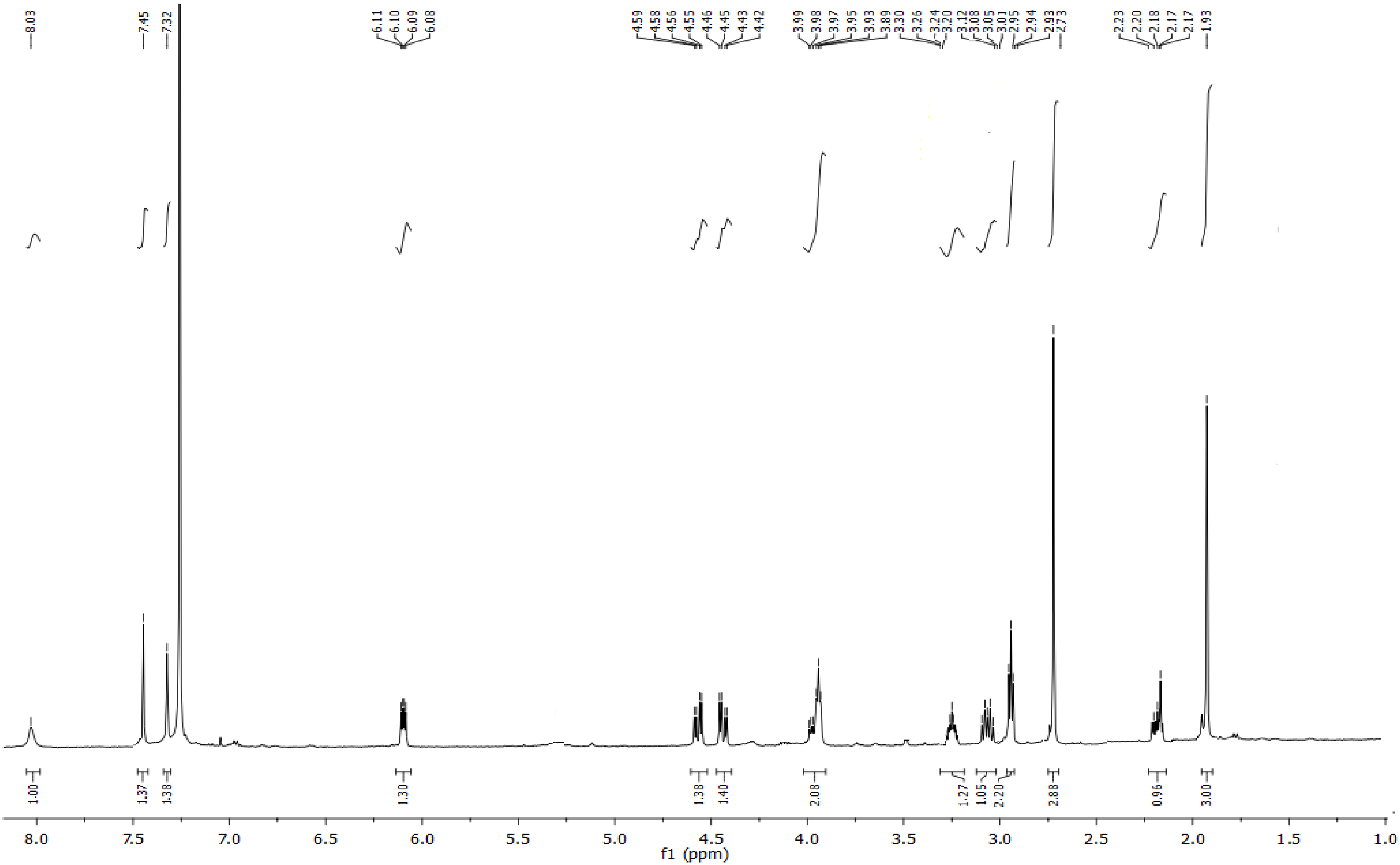

[13] C NMR (126 MHz, CDCl3) of compound **14a**

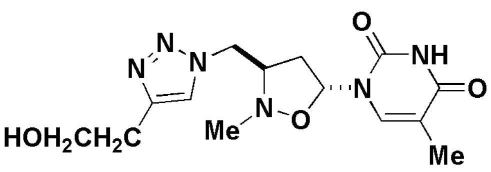

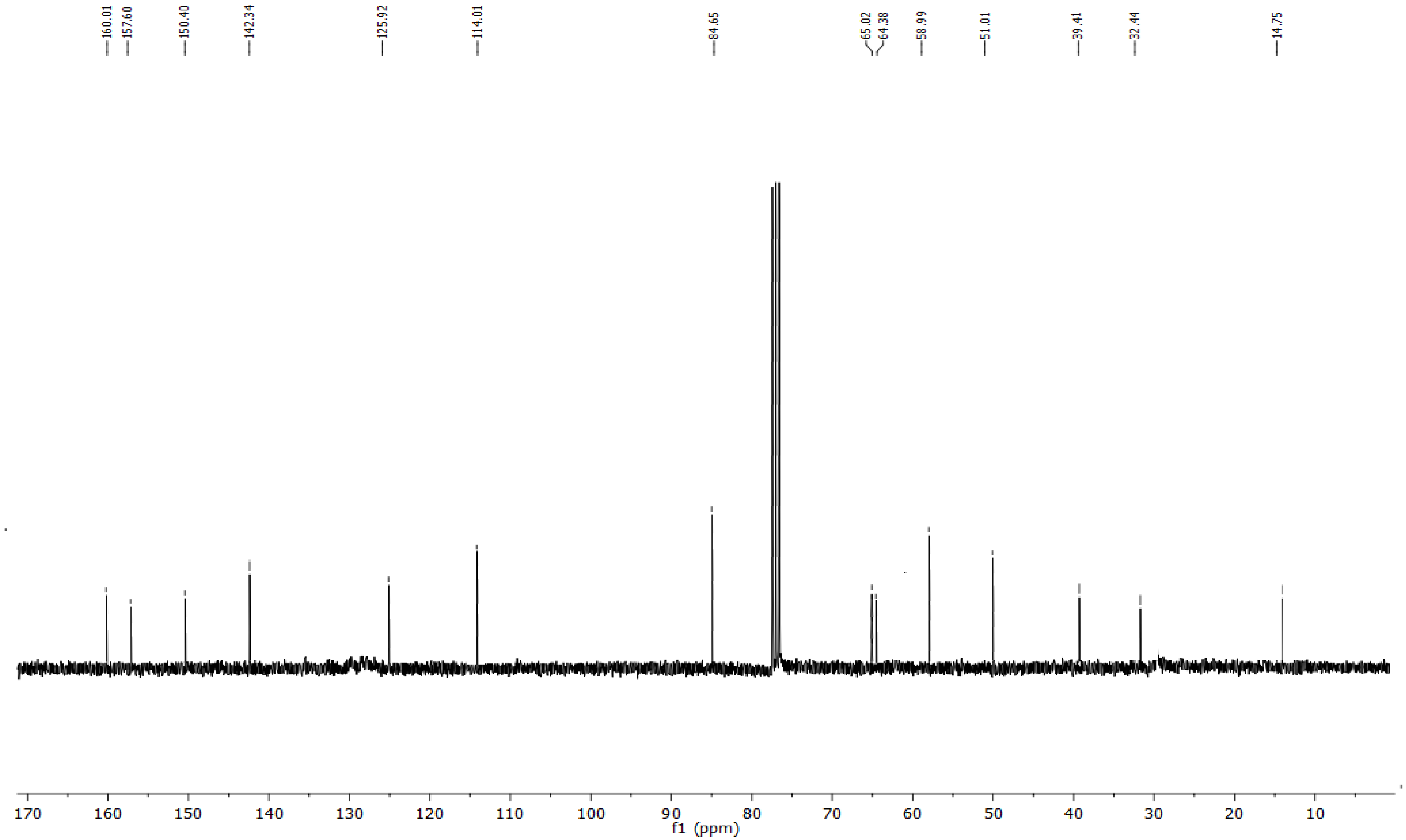

[1] H NMR (500 MHz, CDCl3) of compound **14b**

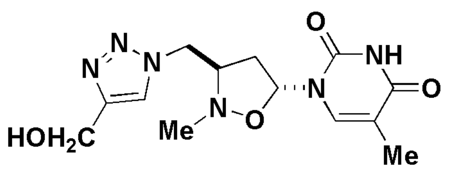

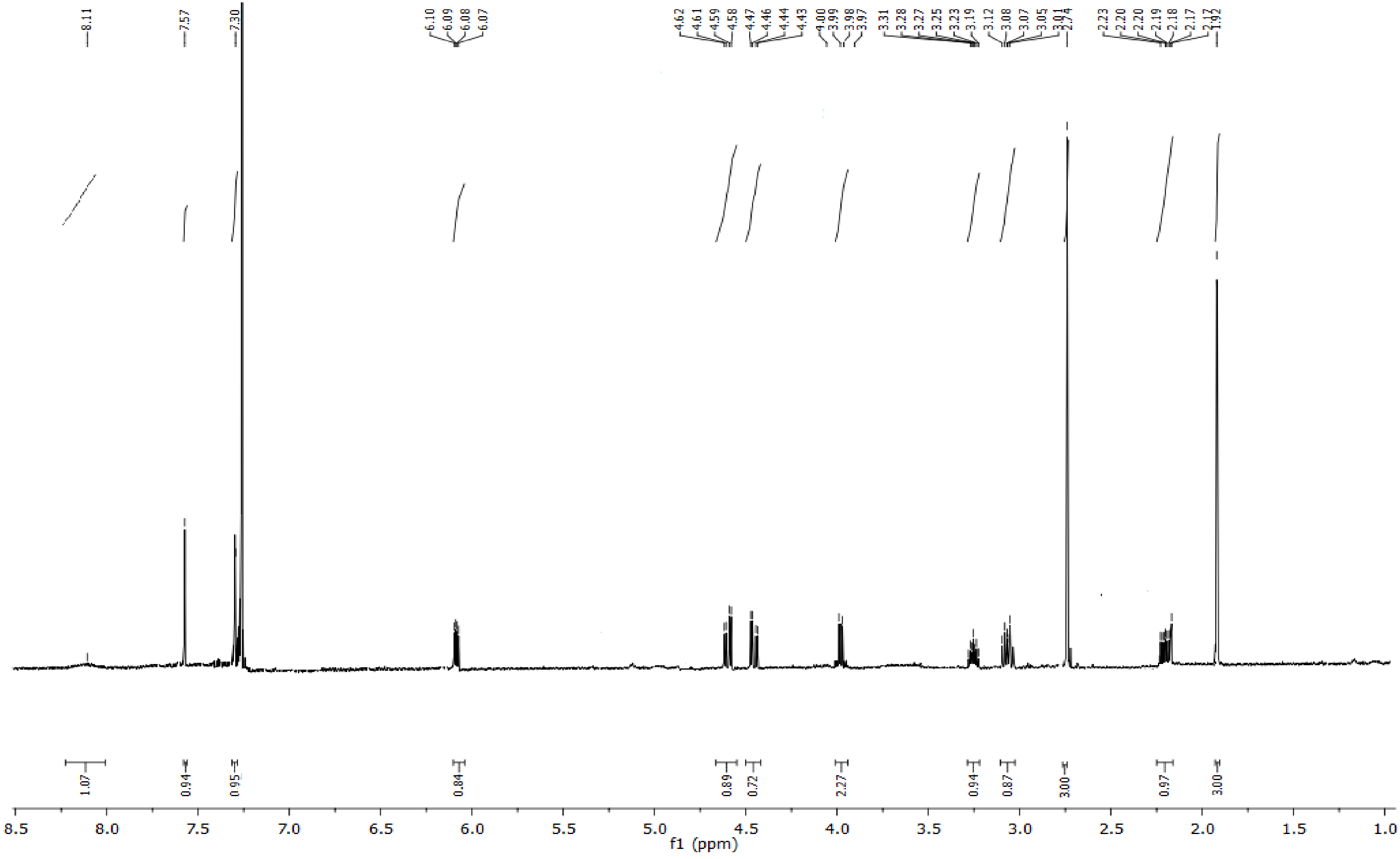

[13] C NMR (126 MHz, CDCl3) of compound **14b**

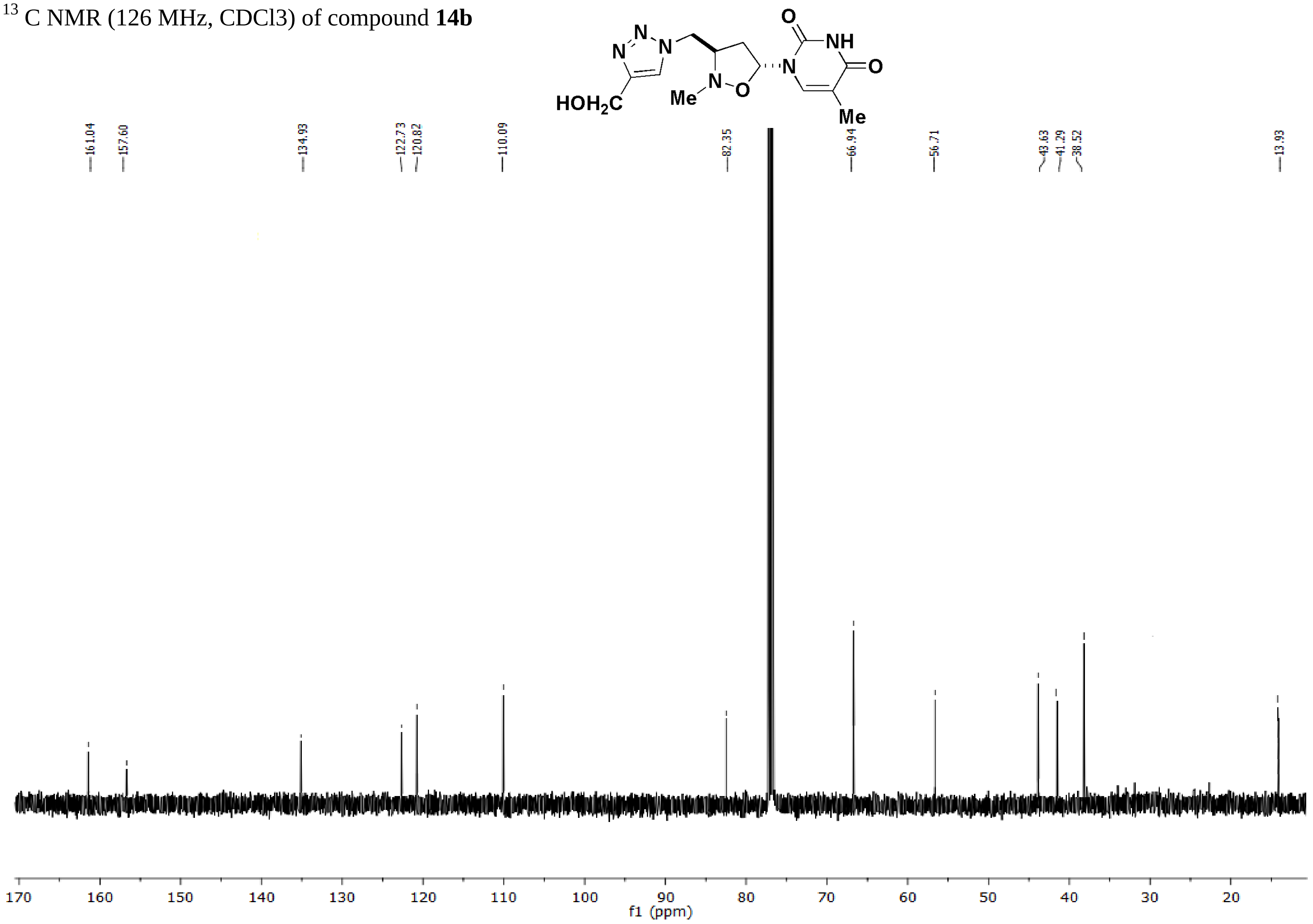

^{1}H NMR (300 MHz, CDCl3) of compound **14c**

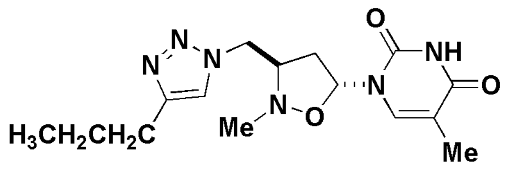

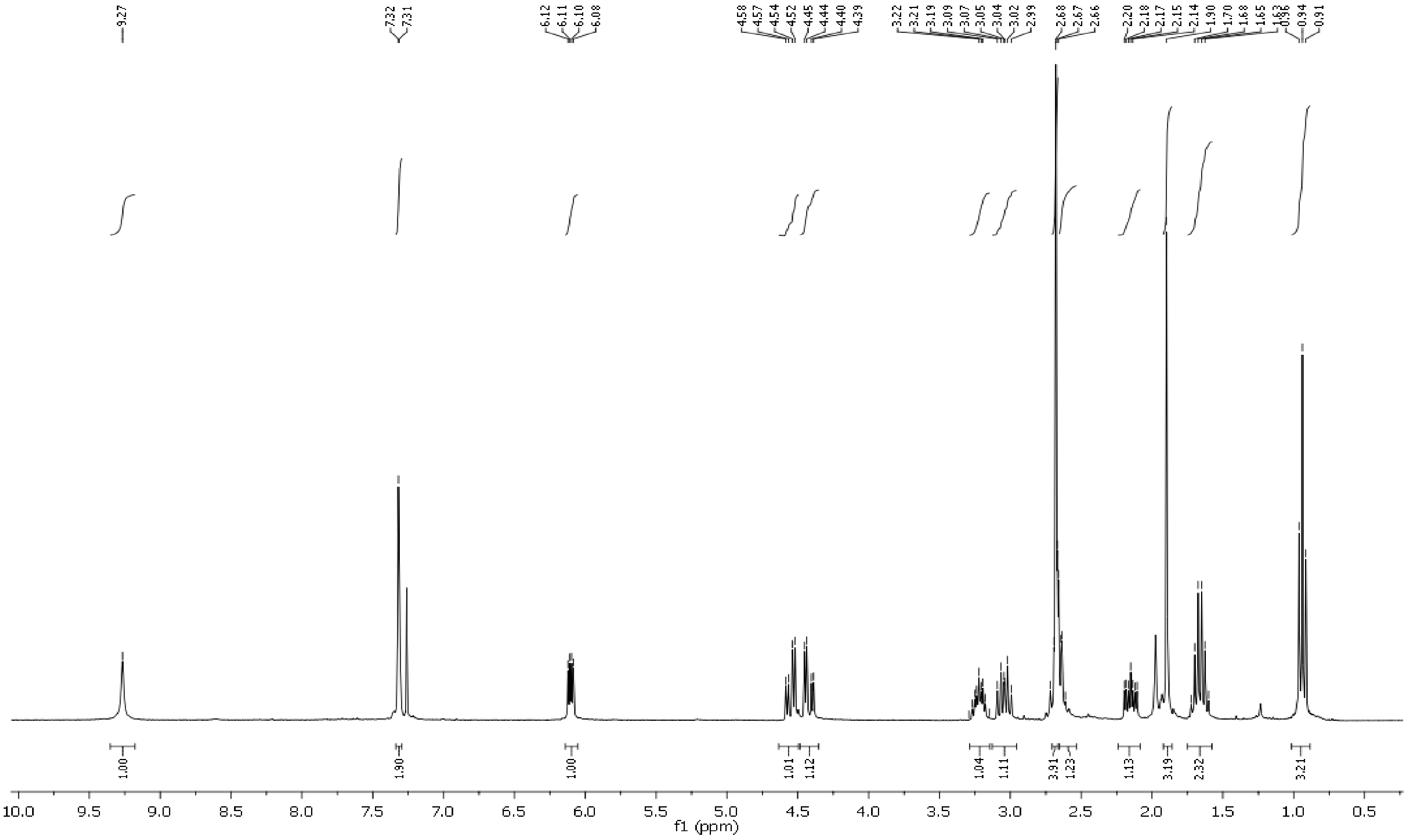

^{13} C NMR (126 MHz, CDCl3) of compound **14c**

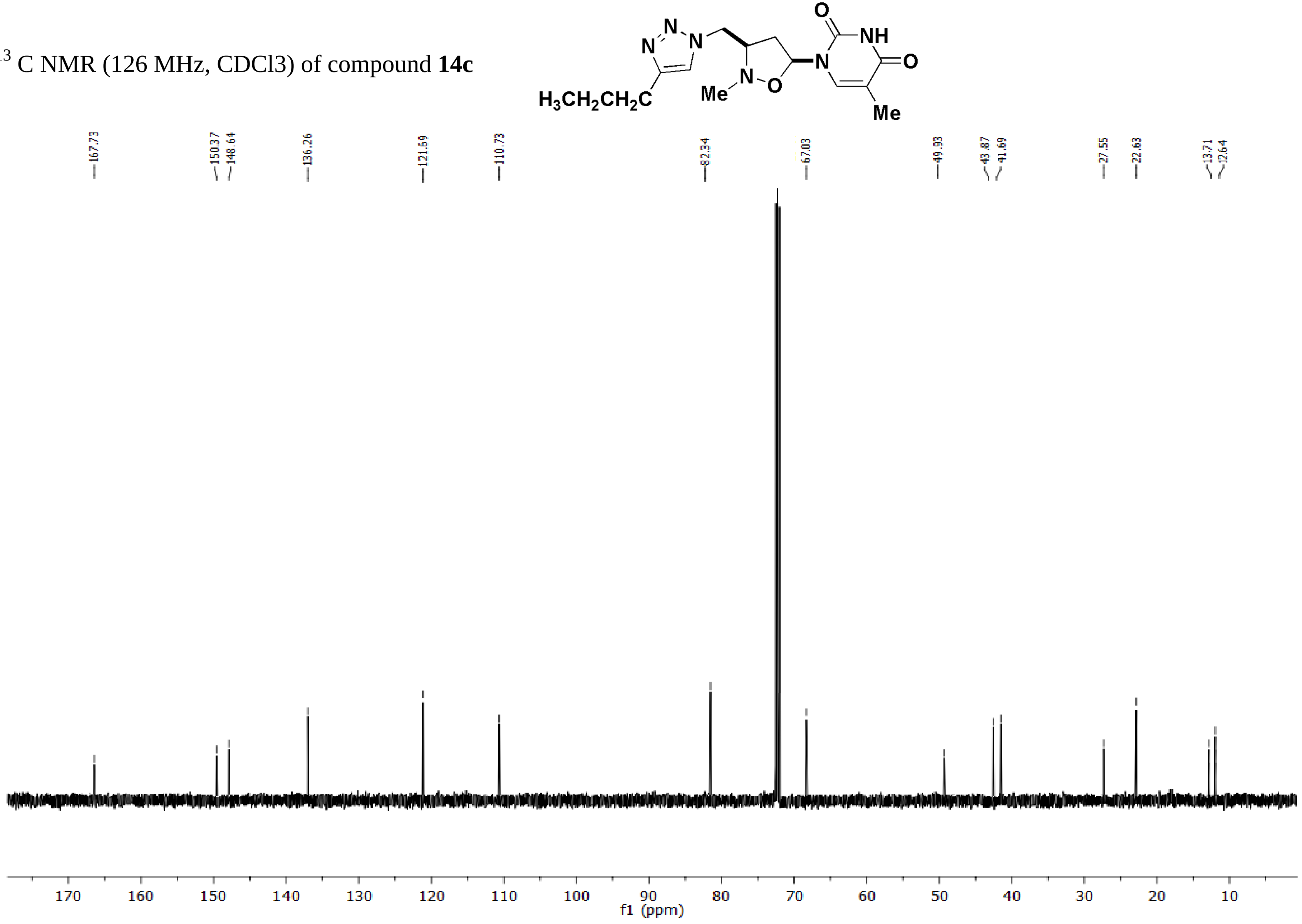

[1] H NMR (300 MHz, CDCl3) of compound **14d**

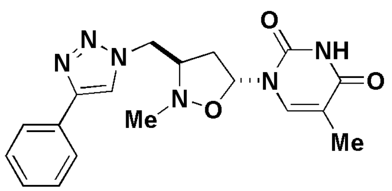

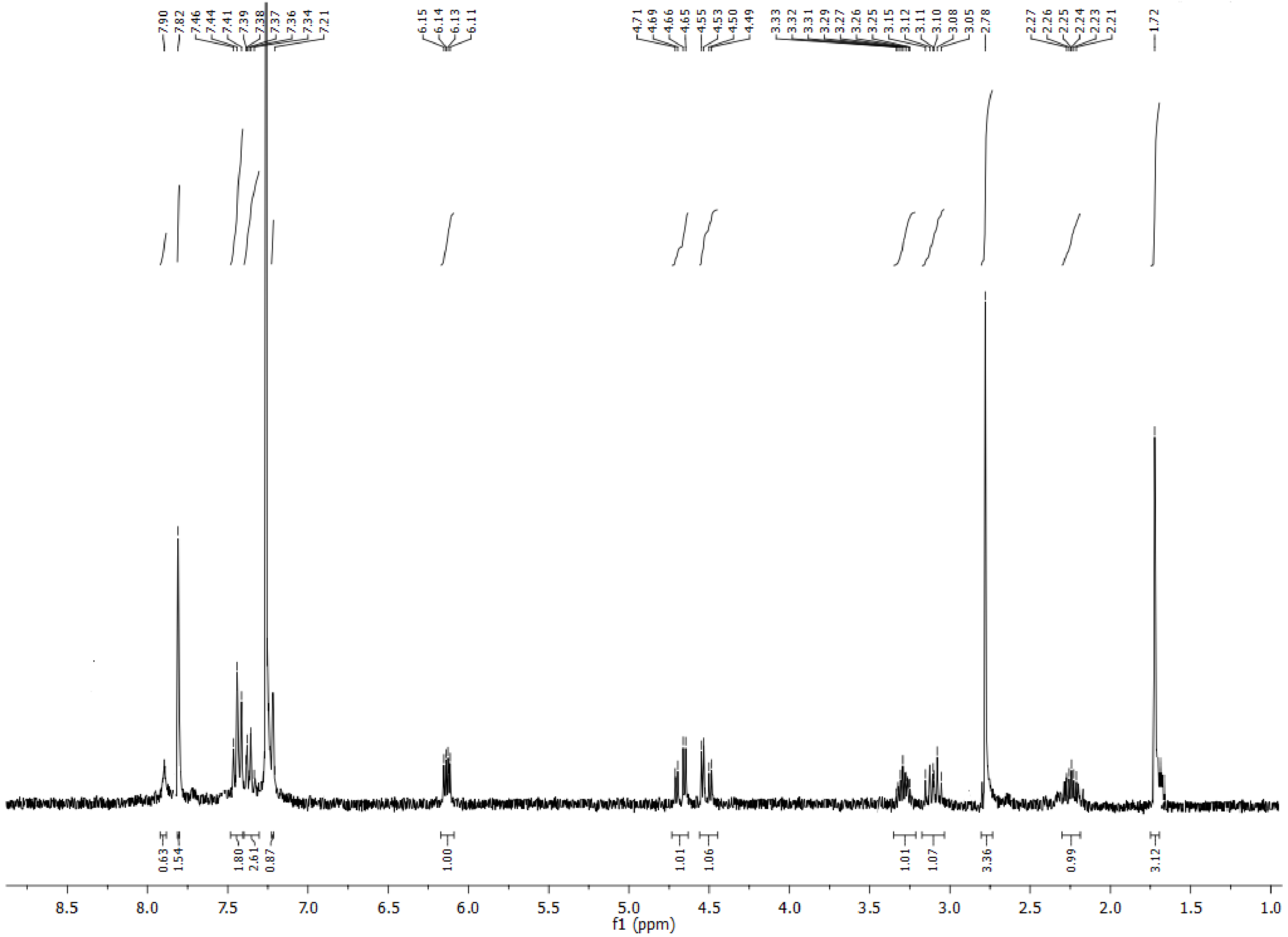

^{13}C NMR (126 MHz, CDCl3) of compound **14d**

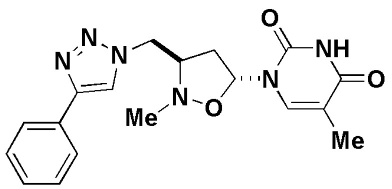

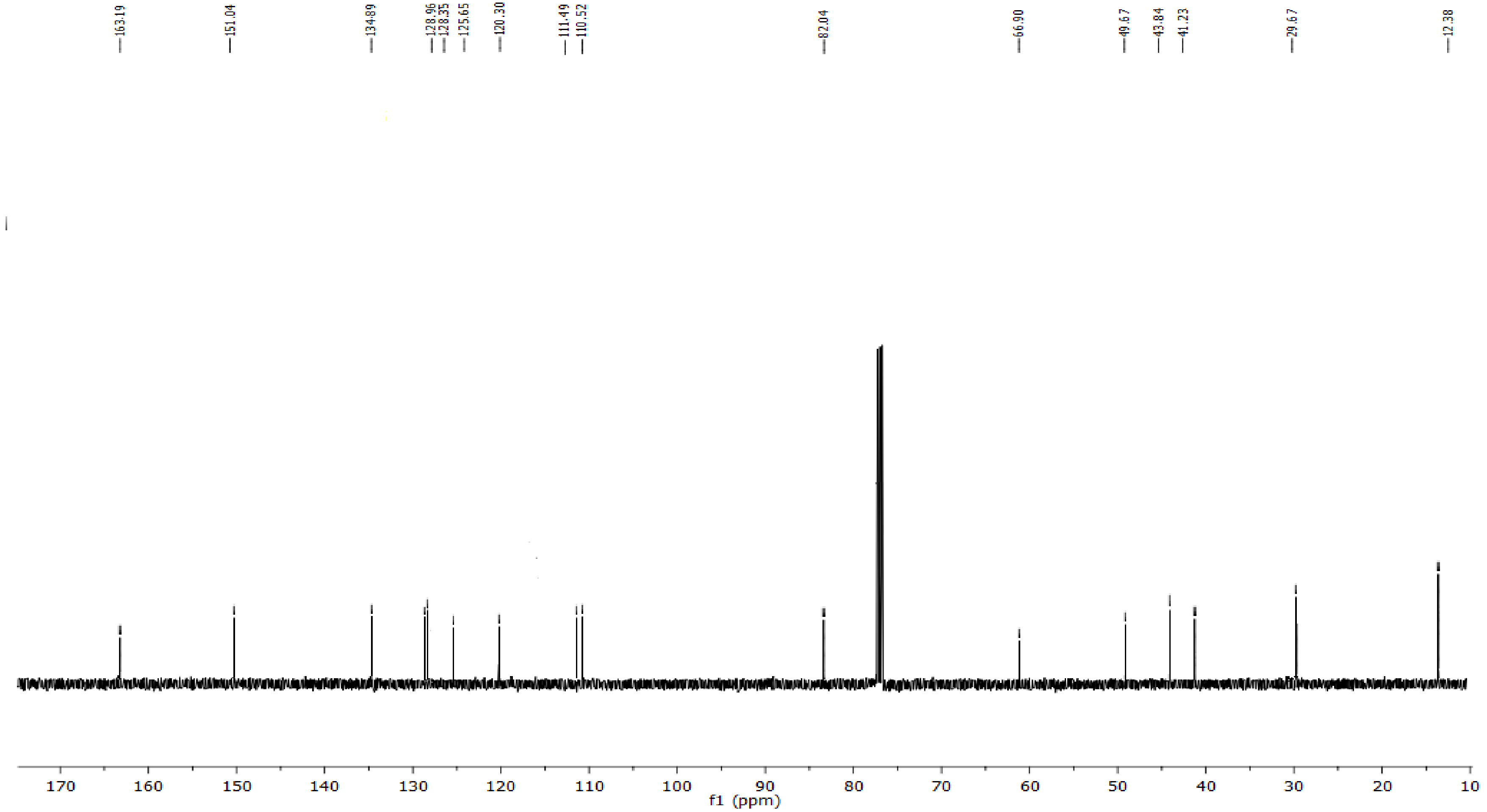

[1] H NMR (300 MHz, CDCl3) of compound **14e**

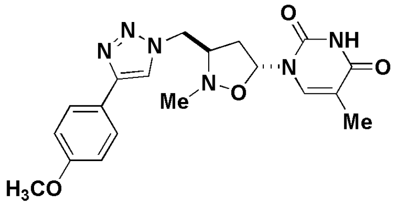

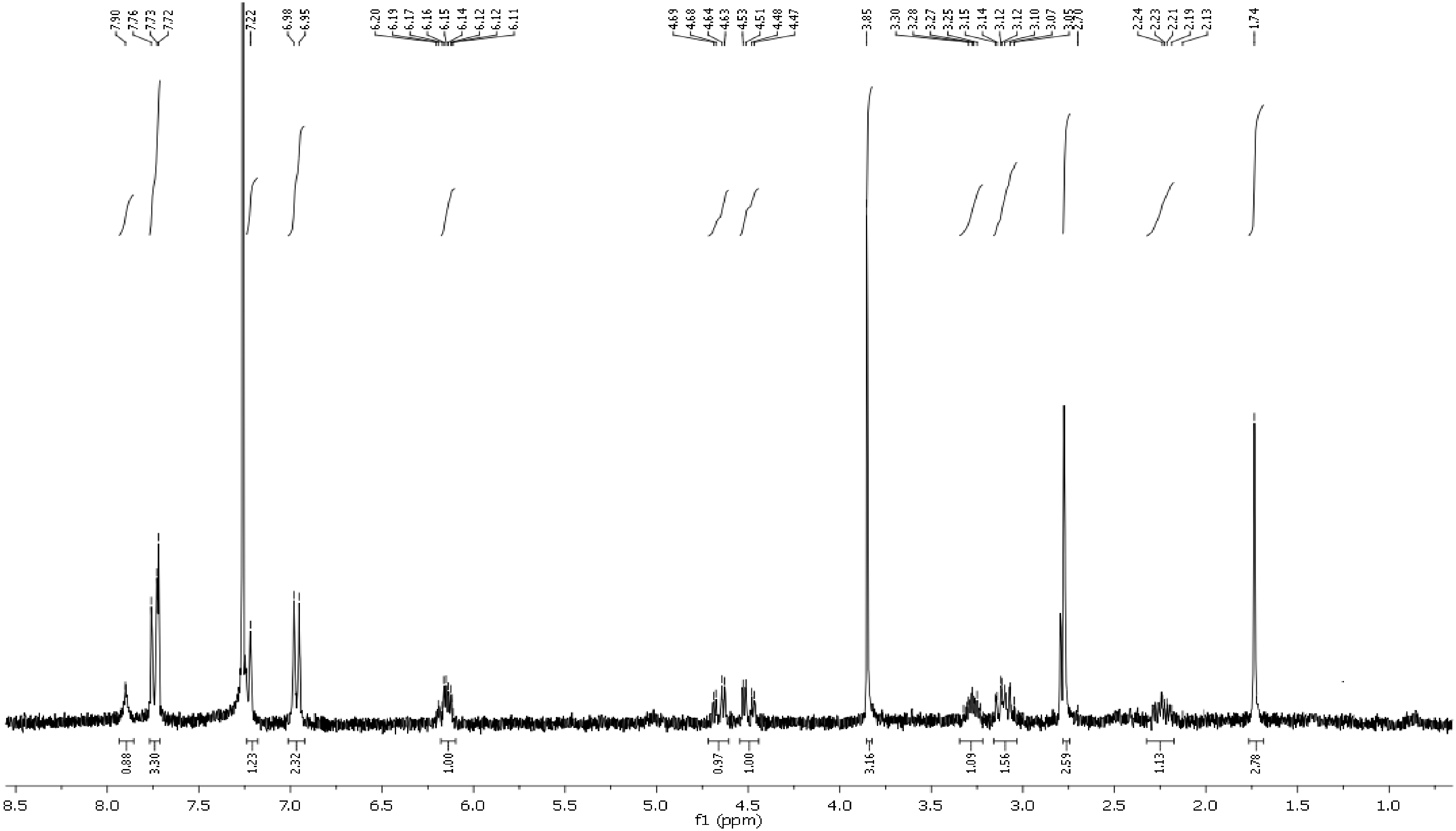

^{13} C NMR (126 MHz, DMSO) of compound **14e**

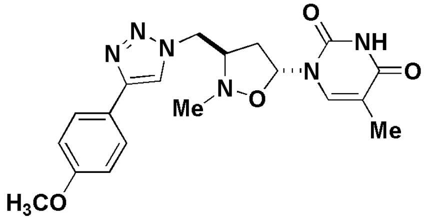

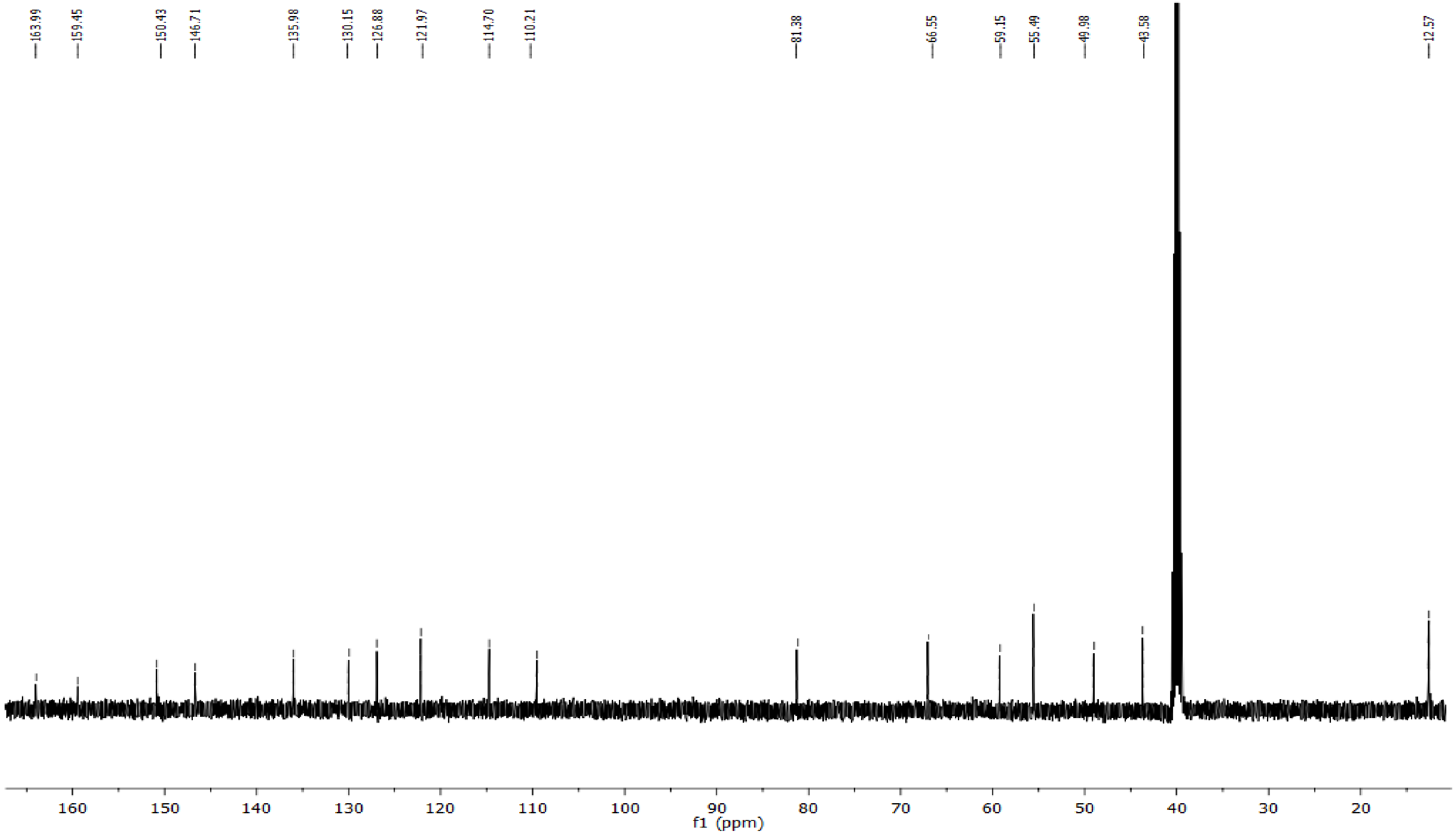

1

H

NMR (500 MHz, DMSO) of compound **14f**

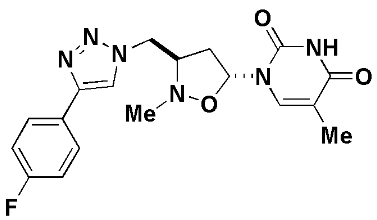

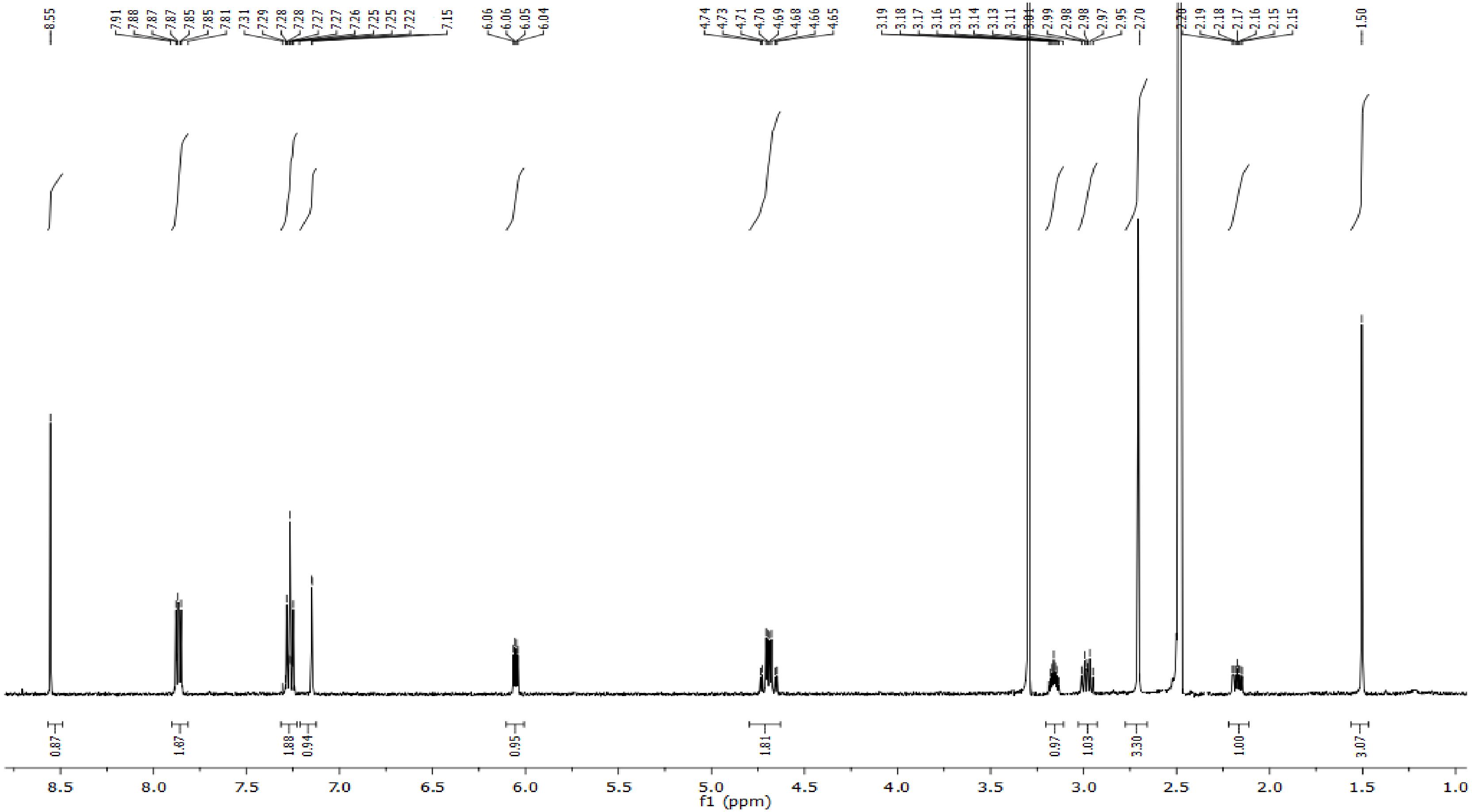

[13] C NMR (126 MHz, CDCl3) of compound **14f**

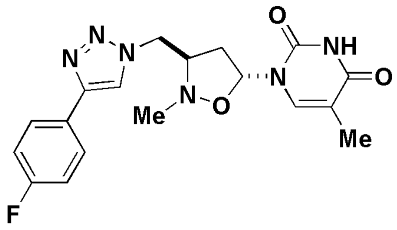

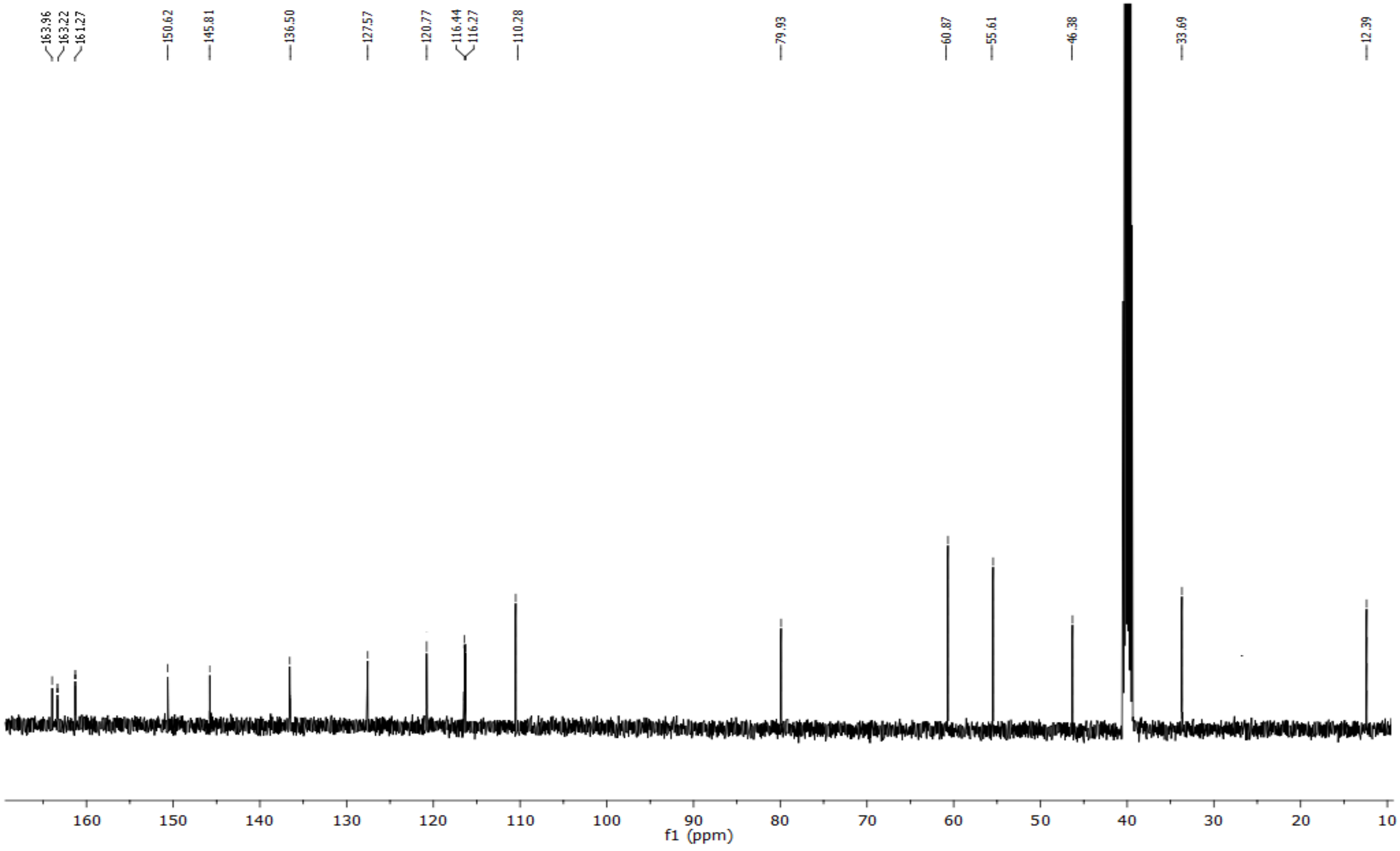

[1] H NMR (300 MHz, CDCl3) of compound **14g**

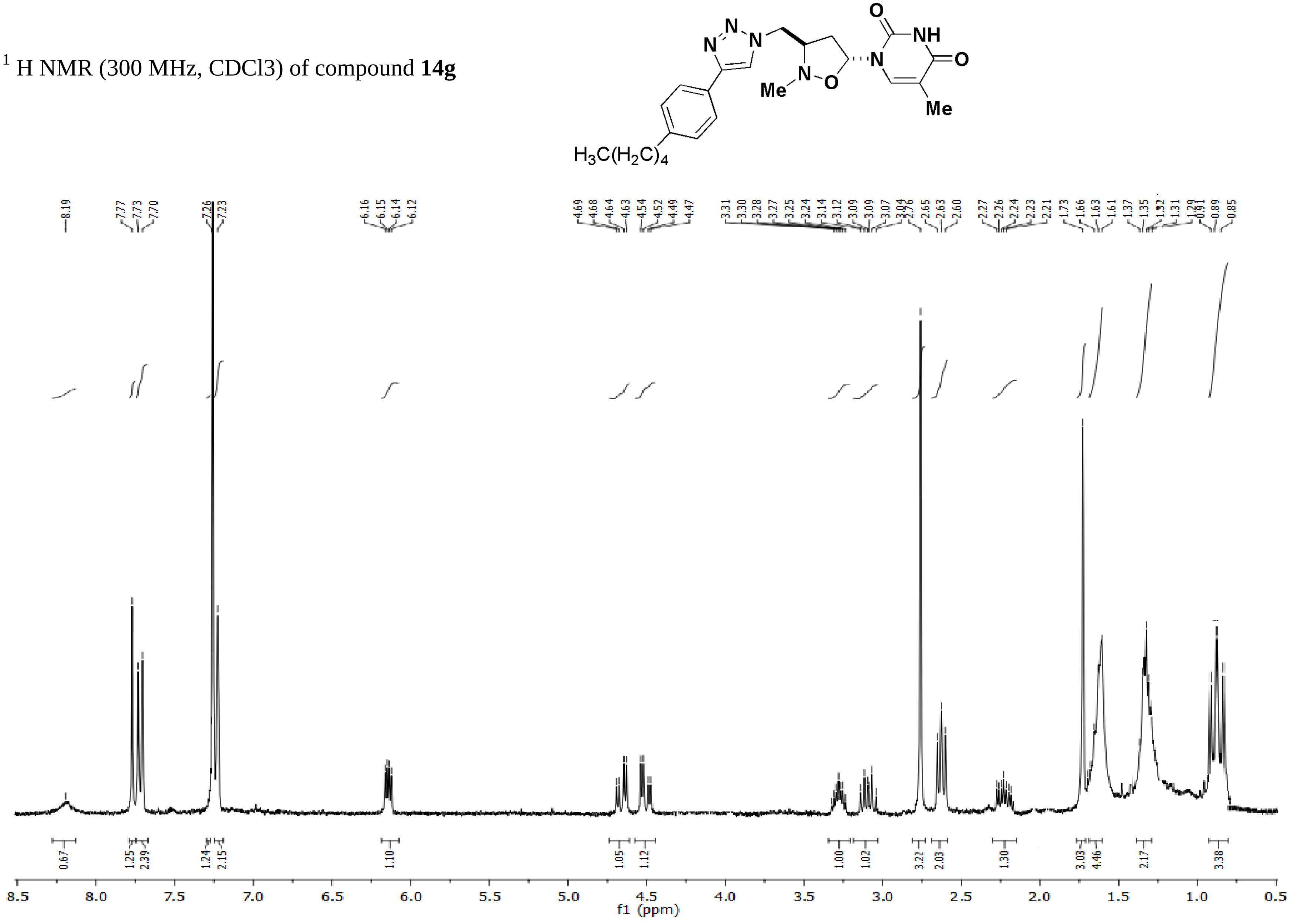

13

C

NMR (126 MHz, DMSO) of compound **14g**

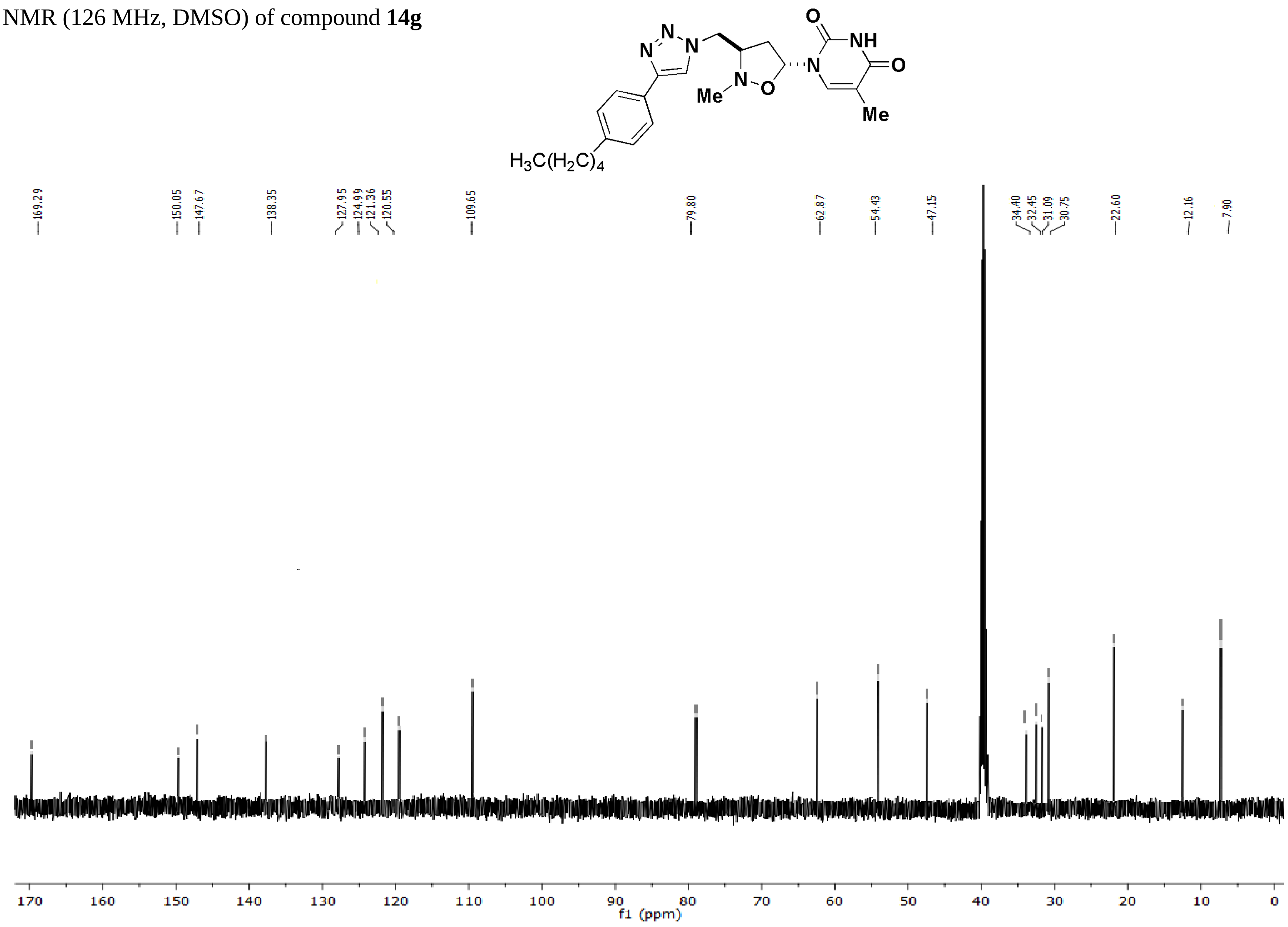

Supplement: File 1 — Preparation and analytical data of compounds 9–14. Copies of 1H and 13C NMR spectra of all new compounds. [file Beilstein_J_Org_Chem-11-328-s001.pdf]
